# Expression of TRX1 optimizes the antitumor functions of human CAR T cells and confers resistance to a pro-oxidative tumor microenvironment

**DOI:** 10.3389/fimmu.2022.1063313

**Published:** 2022-12-14

**Authors:** Emre Balta, Nina Janzen, Henning Kirchgessner, Vasiliki Toufaki, Christian Orlik, Jie Liang, Divya Lairikyengbam, Hinrich Abken, Beate Niesler, Karin Müller-Decker, Thomas Ruppert, Yvonne Samstag

**Affiliations:** ^1^ Section of Molecular Immunology, Institute of Immunology, Heidelberg University Hospital, Heidelberg, Germany; ^2^ Leibniz Institute for Immunotherapy, Division of Genetic Immunotherapy, University Regensburg, Regensburg, Germany; ^3^ Department of Human Molecular Genetics, Heidelberg University Hospital, Heidelberg, Germany; ^4^ Counter Core Facility, Institute of Human Genetics, Heidelberg University Hospital, Heidelberg, Germany; ^5^ Core Facility Tumor Models, German Cancer Research Center, Heidelberg, Germany; ^6^ Mass Spectrometry Core Facility, Center for Molecular Biology, Heidelberg University, Heidelberg, Germany

**Keywords:** cancer immunotherapy, CAR T cells, redox regulation, thioredoxin-1, ROS, tumor microenvironment

## Abstract

Use of chimeric antigen receptor (CAR) T cells to treat B cell lymphoma and leukemia has been remarkably successful. Unfortunately, the therapeutic efficacy of CAR T cells against solid tumors is very limited, with immunosuppression by the pro-oxidative tumor microenvironment (TME) a major contributing factor. High levels of reactive oxygen species are well-tolerated by tumor cells due to their elevated expression of antioxidant proteins; however, this is not the case for T cells, which consequently become hypo-responsive. The aim of this study was to improve CAR T cell efficacy in solid tumors by empowering the antioxidant capacity of CAR T cells against the pro-oxidative TME. To this end, HER2-specific human CAR T cells stably expressing two antioxidant systems: thioredoxin-1 (TRX1), and glutaredoxin-1 (GRX1) were generated and characterized. Thereafter, antitumor functions of CAR T cells were evaluated under control or pro-oxidative conditions. To provide insights into the role of antioxidant systems, gene expression profiles as well as global protein oxidation were analyzed. Our results highlight that TRX1 is pivotal for T cell redox homeostasis. TRX1 expression allows CAR T cells to retain their cytolytic immune synapse formation, cytokine release, proliferation, and tumor cell-killing properties under pro-oxidative conditions. Evaluation of differentially expressed genes and the first comprehensive redoxosome analysis of T cells by mass spectrometry further clarified the underlying mechanisms. Taken together, enhancement of the key antioxidant TRX1 in human T cells opens possibilities to increase the efficacy of CAR T cell treatment against solid tumors.

## Background

The remarkable success of innovative immunotherapeutic approaches in recent years has opened a new era in cancer treatment. In particular, therapies based on tumor specific T cells expressing chimeric antigen receptors for tumor antigens (CAR T cells) are remarkably effective against B cell lymphoma and leukemia, with remission rates of up to 90% in clinical studies (1). Despite these successes, the therapeutic efficacy of CAR T cells against non-hematopoietic solid tumors (e.g., breast cancer) is limited. Immunosuppression in the solid tumor microenvironment (TME) is a central reason for the failure of CAR T cell therapies (2).

The solid TME includes immunosuppressive immune cell infiltrates, non-immune cell infiltrates, and soluble molecules (reviewed in [3)]. A major cause of tumor-mediated immunosuppression is the pro-oxidative milieu characterized by relatively high levels of reactive oxygen species (ROS), which have various intracellular and extracellular sources. ROS are generated endogenously as byproducts of mitochondrial ATP production *via* the electron transport chain, from endoplasmic reticulum, or specifically by enzymes, such as nicotinamide adenine dinucleotide phosphate (NADPH) oxidases. Apart from tumor cells, other cells in the TME (stromal cells, tumor-infiltrating neutrophils, macrophages, myeloid-derived suppressor cells, and endothelial cells) produce ROS, contributing to the pro-oxidative milieu. Excessive ROS are also generated by tumor metabolic reprogramming and hypoxic conditions [reviewed in (4, 5)]. Many cells in the TME die due to low O_2_ and nutrient levels or by tumor-specific lymphocyte killing, which also potentially leads to release of hydrogen peroxide (H_2_O_2_) and other ROS radicals [reviewed in (6)]. Conventional anticancer treatments, such as chemotherapy or radiation therapy, directly and indirectly cause further ROS accumulation in the TME (7).

Cells usually maintain ROS equilibrium *via* antioxidant systems, including detoxification enzymes, oxidoreductases, and the glutathione system. Among these, thioredoxins (TRXs) are a family of proteins containing a TRX fold that catalyze oxidoreductase reactions by a dithiol-disulfide exchange mechanism involving two redox-active cysteines (CxxC motif) that reduce target (oxidized) proteins and oxidize TRX1. TRX1 gets reduced by NADPH in a reaction catalyzed by thioredoxin reductase I (TRXR1). This system is highly upregulated in tumor cells and positively associated with tumor cell metastatic potential (8). Glutaredoxins (GRXs) are another type of oxidoreductase primarily responsible for deglutathionylation of S-glutathionylated proteins, which contributes to glutathione pool homeostasis and to reduced protein states, thereby controlling various redox signaling networks. While the absence of GRXs is associated with several diseases (9), no direct correlation of GRX expression with tumor progression (as observed for the TRX1 system) has been identified. On the contrary to the tumors, the importance of TRX1 or GRX1 in human T cell responses largely remained elusive. In this context, in murine T cells, deletion of Thioredoxin 1 Reductase (TRXR1) was shown to be indispensable for the development of thymocytes. Furthermore, the authors showed that TRXR1 is critical for nucleotide biosynthesis in activated murine T cells (10). Another study revealed an upregulation of TRX1 and potentially higher TRX1 activity due to Thioredoxin-interacting protein (TXNIP) downmodulation in NK cells, when the cells were cultured with IL-15 (11). These findings provide indirect evidence that TRX1 may be critical for human T cell responses.

ROS influence T cell activity through oxidative modifications that regulate cellular processes by acting on protein thiols, DNA, and lipids. In small quantities, ROS (particularly H_2_O_2_) are important signal carriers (12, 13); however, excess ROS induce oxidative stress and cell damage, primarily when the buffering capacity of antioxidant systems is exceeded. Oxidative stress leads to hypo-responsiveness, or even death, of T cells, at least partly *via* oxidation, and thereby inactivation of the actin-remodeling protein cofilin (14–16), leading to actin cytoskeleton stiffening and impaired T cell migration (15). Tumor-specific T cells require a highly dynamic actin cytoskeleton to infiltrate tumors. The fact that T cells often accumulate in the tumor periphery, but are unable to infiltrate the tumor parenchyme may be partly due to ROS-induced actin cytoskeleton dysfunction. The actin-bundling protein L-plastin (LPL) is also oxidized under pro-oxidative conditions and contributes to reduced cell migration  (17). Overall, ROS produced in the TME are likely key to diminished T cell infiltration into tumors.

ROS-induced T cell hypo-responsiveness can occur at several stages. Hence, understanding the influence of ROS on T cells at the molecular level and strengthening T cell resistance to ROS is a promising approach to improve CAR T cell-based therapies against solid tumors. The aim of this study was to investigate the potential of antioxidant systems to influence CAR T cell antitumor efficacy and persistence under pro-oxidative conditions.

## Methods

### Cell culture

Human peripheral blood mononuclear cells were purified by Ficoll-Hypaque-based density gradient centrifugation of heparinized blood from healthy volunteers. Resting human peripheral blood T cells (PBT) were purified *via* negative magnetic bead selection with a Pan T Cell Isolation Kit, according to the manufacturer’s instructions (Miltenyi Biotec, 130-096-535). This study was approved by the Ethics Committee of Heidelberg University (S-089/2015).

SKBR3 (DMSZ, ACC736) and MDA-MB-453 (DMSZ, ACC 65) tumor cells were purchased from the German Collection of Microorganisms and Cells (DSMZ). Human embryonic kidney (HEK293T) cells were cultured in Dulbecco’s modified Eagle medium (DMEM) supplemented with 10% fetal calf serum (FCS) and 4 mM L-glutamine. CAR T cells were cultured in X-Vivo 15 cell culture media (Lonza) supplemented with 40 U/ml IL-2, 10 ng/ml IL-7, and 10 ng/ml IL-15. All cells were maintained at 37°C with 5% CO_2_. Cell lines were routinely tested for Mycoplasma.

### Intracellular ROS detection

200,000 cells were untreated or treated with 100 µM H_2_O_2_ in 100 µl RPMI + 0.5% FCS for 15 min. Then, samples were washed and stained with 5 µM CM-H_2_DCFDA ROS sensor (Thermo Fischer Scientific, C6827) in 100 µl phosphate-buffered saline (PBS) for 15 min. ROS sensor fluorescence intensity was detected at excitation wavelength, 485 nm, and emission wavelength, 535 nm using multicolor flow cytometry (LSR II; BD Biosciences).

### siRNA knockdown

PBTs were nucleofected with control (5’-GGCATTCCAGAGGATGGTAAT-3’) or TRX1-specific (5’-GCTTCAGAGTGTGAAGTCAAA-3’) siRNA (Amaxa Nucleofector II device, Lonza). Four hours later, 7.5 × 10^6^ cells were transferred to each well of a 12-well plate precoated with 20 ng/ml anti-CD3 (BD Biosciences, 566685) and 1 µg/ml anti-CD28 antibodies (BD Biosciences, 555725). Cells were cultured in complete RPMI medium for up to 3 days. Western blotting and flow cytometry were used to validate knockdown of endogenous TRX1.

### Cell proliferation

Ten million PBTs were stained with 1 µM CFSE (Thermo Fischer Scientific) in 1 ml PBS for 15 min. Then, samples were washed twice with PBS by centrifugation at 300 x g. Next, 5 × 10^6^ CFSE-stained PBTs were nucleofected with 20 µM siTRX1 or siControl (Horizon Discovery), using the X001 program. Cells were then cultured in complete RPMI medium for 4 h, and subsequently transferred to 96-well flat bottom plates coated with anti-CD3 and anti-CD28 antibodies (5 × 10^4^ cells per well), and cultured in 200 µl complete RPMI for 3 days. Loss of fluorescence intensity in dividing cells was assessed daily by flow cytometry (LSR II, BD Biosciences). Cell division index values were calculated using FlowJo software according to the manufacturer’s instructions.

### Immunoblotting

Equal amounts of cell lysates were separated on sodium dodecyl sulfate (SDS)-polyacrylamide gels and transferred to polyvinylidene fluoride membranes. Then, membranes were stained with indicated primary antibodies, as follows: mouse anti-human TRX1 (1:500, BD Biosciences, 559969), rabbit anti-human GLRX1 (1:1000, R&D Systems, AF3399), rabbit anti-human actin (1:1000, Sigma, A2066), or mouse anti-human GAPDH (1:2000, Ambion, 4300), followed by staining with IRDye 680 or IRDye 800-labeled secondary antibodies (1:10,000, LI-COR Biosciences). Membranes were then scanned using a LI-COR Odyssey scanner (LI-COR Biosciences) (18).

### Cloning of CAR constructs

Retroviral cDNA constructs encoding for ctrl-CAR or HER2-CAR were kindly provided by Rolf Kiessling (19). The cDNA constructs were then cloned into pLJM1-eGFP lentiviral vectors (Addgene, 19319) using restriction-ligation based cloning. For that purpose, primers which are N-terminally flanked by the EcoRI or NheI restriction enzyme cutting sites were employed. To generate antioxidant expressing HER2-CAR constructs, initially cDNAs of TRX1 and GRX1were cloned into the plJM1-eGFP vector. For cloning of TRX1 and GRX1 the respective cDNAs were purchased from Dharmacon.

### Lentivirus production

Lentivirus production, titration, and target cell transduction were performed according to standard protocols. Briefly, 6 × 10^6^ HEK293T cells were seeded into 150-mm culture dishes. The next day, expression constructs, pMD2.G (Addgene, 12259) and psPAX2 plasmids (Addgene, 12260) were mixed at a ratio of 4:3:1 in 500 µl OptiMEM™ medium (Thermo Fischer Scientific, 31985062), and the DNA mixture then mixed with polyethylenimine (PEI) (Sigma, 765090) at a 2:1 PEI : DNA ratio. PEI-DNA complexes were incubated for 20 min at room temperature and added onto HEK293T cells dropwise. Cells were incubated overnight under standard cell culture conditions. The next day, culture medium was replaced with fresh complete DMEM. Cells were incubated for 2 days before supernatants were collected to harvest viruses by centrifugation (400 x *g*, 5 min) to remove cell debris. Cleared lysates were filtered through 0.45-µm pore-size filters (Merck Millipore, Z290793), and viral particles concentrated by ultracentrifugation (100,000 x *g*, 90 min). Viruses were aliquoted and stored at –80°C until use.

### CAR T cell production

CAR T cells were generated according to conventional lentiviral transduction protocols. Briefly, PBTs were activated using Dynabeads Human T cell activator CD3/CD28 (Thermo Fischer Scientific, 11161D) beads for 1 day. Then, cells were mixed with lentiviral vectors at a multiplicity of infection of 10. Next, cells were spinoculated (30 min, 300 x *g*, room temperature) to increase the transduction efficiency, then cultured overnight in X-vivo 15 medium supplemented with 10 ng/ml IL-7 (Peprotech, AF-200-07), 10 ng/ml IL-15 (Peprotech, 200-15), 40 U IL-2 (Peprotech, AF-200-02), and 8 µg/ml polybrene (Sigma-Aldrich, TR-1003). One day after transduction, an equal amount of X-vivo 15 medium was added to the cell cultures. Four days after transduction, pseudoviral particles were removed by extensive washing. Samples were maintained in complete RPMI medium supplemented with 10 ng/ml IL-7 and 10 ng/ml IL-15 and 20 U/ml IL-2 until further use. Expression of the CAR receptor on T cells was assessed by flow cytometry using PE-conjugated goat anti-human immunoglobulin G (IgG) antibody (Novus Biologicals, NBP1-75002PE).

### Immune synapse formation

MDA-MB-453 tumor cells (1.5 × 10^6^) were seeded into T75 flasks 1 day prior to coculture to generate single cells. The next day, CAR T cells were counted and treated with 100 μM H_2_O_2_ for 30 min as described above. Meanwhile, MDA-MB-453 cells were detached using PBS + 5 mM EDTA for 3 min. Next, CAR T cells and MDA-MB-453 cells were washed with complete RPMI medium. Then, 5 × 10^5^ CAR T cells and 5 × 10^5^ MDA-MB-453 cells (ratio, 1:1) were mixed in round-bottom polystyrene tubes in 100 μl complete RPMI for 15 and 45 min, followed by gradual addition of 1.5 ml 1.5% paraformaldehyde (PFA) onto the samples while they were gently vortexed to prevent non-specific interactions. Samples were fixed using 1.5% paraformaldehyde for 10 min at room temperature, followed by permeabilization in FACS Wash Saponin (FWS) buffer (PBS + 1% bovine serum albumin [BSA] + 0.1% saponin) for 10 min. Finally, samples were stained with either HER2-PE (1:500, R&D Sciences, FAB1129P), CD3-phycoerythrin (PE)-Cy7 (1:50, BioLegend, 300420), CD8-PE-Cy5 (1:4000, BD Biosciences, 555368), Granzyme B-fluorescein isothiocyanate (FITC) (1:10, BD Biosciences, 561998), Perforin-allophycocyanin (APC) (1:50, BioLegend, 308112), and DAPI (1:10,000, Sigma, D9542),; or HER2-PE, CD3-PE-Cy7, LFA1-PECy5 (1:100, BD Biosciences, 555512), DAPI, Granzyme A-FITC (1:20), antibody mixtures and SiR-Actin (Tebu-Bio, SC001) stain solutions.

### Analysis of immune synapse formation

Cells (25,000 per sample) were measured using an ImageStream instrument (Mark II, Amnis), then analyzed using Ideas 6 software. Briefly, cells that were out of focus in the brightfield image were gated out using the “Gradient RMS” feature. Then, DAPI-stained cells were further separated based on HER2 and CD3 signals. The HER2/CD3 double-positive population were considered potential couples. Then, a set of masks was generated to quantify the cytolytic immune synapses. Cell clumps were removed using the area of DAPI (Area_M07) and aspect ratio of CD3-positive cells (Aspect Ratio Intensity_M06_Ch6) features. Then, a synapse mask was generated using the CD3 signal (Morphology_M06), HER2 signal (Morphology_M03), and Valley mask (ValleyM07, Ch07,3)). Using the area of synapse mask (Area_Synapse mask) and area of Morphology Ch3 (Area_Morphology (M03, Ch03) (True synapses)), followed by area of synapse mask (Area_Synapse mask) and area of Morphology Ch6 [(Area_Morphology (M06, Ch06)) (Synapse area_Corrected)], genuine synapses were identified.

To quantify the degree of protein translocation into the interaction zone in the T cell side, a fill mask was generated based on CD3 staining, to define T cells (Fill (M06)). To identify the T cell side of synapses, Fill mask (M06) was combined with synapse mask (Fill (M06 and Synapse mask). Then the intensities of different proteins (CD3, LFA-1, Granzyme A, Granzyme B, Perforin) in T cells and in the T cell-side of the immune synapse were calculated using the intensity feature. Next, the percentage protein enrichment was calculated using the following formula:

Percent protein enrichment = (Intensity of protein at the T cell side/Intensity of the protein in T cells) × 100

A threshold of 30% was used to identify cells with strong enrichment into immune synapses; granzyme A, granzyme B, and perforin enrichment were calculated in cells meeting this threshold.

### Time-lapse microscopy

SKBR3 tumor cells (30,000) were seeded on 8-well chambers (IBIDI, 005479) and allowed to adhere for 45 min. Next, tumor cell nuclei were stained using 1:100 diluted DAPI (Sigma, D9542) live-cell staining solution. Cells were washed extensively using PBS, then stained with 10 µM caspase 3/7 sensor (Thermo Fischer Scientific, C10427) in 250 µl PBS for 30 min. Samples were washed extensively and maintained in complete RPMI medium without phenol red. Meanwhile, CAR T cells were either untreated or treated with 25 µM H_2_O_2_ for 1 h, as described above. Then, 1 × 10^5^ CAR T cells were stained with 100 nM Lysotracker deep red (Thermo Fischer Scientific, L7528) in 100 µl complete RPMI for 30 min. Samples were then washed extensively using PBS. Finally, CAR T cells were transferred onto tumor cells, prior to imaging. Time-lapse imaging was performed for 4 h with 5-min intervals, followed by 30-min time intervals for 24 h, using a confocal laser scanning microscope (Nikon, 20x). Three *xy* focal planes per sample were imaged for each time interval.

### Microscopy-based cytotoxicity assay

A microscopy-based cytotoxicity assay was established to analyze HER2-CAR T cell function. Briefly, HER2-positive SKBR3 tumor cells were pre-stained with eF670 (Thermo Fischer Scientific, 65-0840-85) for 15 min. Then, 3 × 10^4^ SKBR3 cells were allowed to adhere on 10-mm coverslips in 48-well plates overnight. The next day, control (Ctrl-CAR), HER2-, or antioxidant-enhanced HER2-CAR T cells were untreated or treated with the indicated H_2_O_2_ concentrations for 1 h. Next, cells were washed and mixed with the indicated ratios of SKBR3 tumor cells in complete RPMI medium and incubated for 8 h and 24 h. Finally, supernatants were collected, aliquoted, and stored at –20°C for further use; remaining adherent cells were fixed, stained with DAPI and phalloidin AF488 (F-actin) (Thermo Fischer Scientific, A12379), and imaged by confocal microscopy as described above. A large scan of 3 x 3 in *xy* dimensions was acquired using a 20x objective, corresponding to imaging of 9 focal planes per sample. The numbers of cells/focal plane were counted using automated ROI analysis based on DAPI and eF670 signals.

Collected supernatants were used for LDH release assays to assess CAR T cell cytotoxicity toward tumor cells and for CAR T cell cytokine release measurements.

### LDH release assay

Supernatants from CAR T cells cultured alone or cocultured with SKBR3 tumor cells were collected for analysis of LDH release using the CyQUANT LDH cytotoxicity assay, according to the manufacturer’s protocol. SKBR3 cells were seeded into 48-well plates as described above. The next day, control CAR T cells (Ctrl-CAR) or HER2-CAR T cells were added at the indicated ratios for 8 h and 24 h. Then, plates were centrifuged at 250 x *g* for 3 min, and 100 μl of supernatant collected and transferred to 96-well flat-bottom plates in duplicate. Next, 50 μl of the reaction mixture was added to each well. Samples were mixed and incubated at RT for 30 min. Then 50 μl stop solution was added to each well and absorbance measured at 490 nm and 680 nm using a plate reader (Tecan, Sunrise Elisa Reader). To determine LDH activity, the 680 nm absorbance value was subtracted from that at 490 nm.

### Repeated antigen exposure assay

HER2-positive SKBR-3 cells were seeded into a 48-well plate (50,000 per well) overnight. The next day, CAR T cells were adjusted to 4 × 10^5^/ml. The medium in the SKBR3 overnight cultures was removed, and 2 × 10^5^ CAR T cells were added in 500 µl medium.

After two days of coculture, 750 µl complete RPMI was added. On day 5, samples were harvested. Briefly, cells were mixed carefully and 200 µl of culture were taken to estimate CAR T cell numbers. Samples were directly stained using CD3-FITC (BioLegend, 344804) antibody, 7AAD (BD Biosciences, 420404), and a defined bead solution (BD Biosciences, 51-90-9001291) in a total volume of 20 µl, incubated at room temperature for 20 min, then directly measured using an LSR II flow cytometer.

### Cytokine release assays

Supernatants from CAR T cells maintained alone or cocultured with SKBR3 tumor cells for 8 and 24 h under control and pro-oxidative conditions were collected as described above. The release of 13 cytokines was analyzed using the LEGENDplex™ Human CD8/NK Panel (13-plex) kit (BioLegend, 740267) according to the manufacturer’s instructions. The same supernatants were also used to detect the release of IFN-γ, using an IFN-γ ELISA kit (Thermo Fischer Scientific, EHIFNG), according to the manufacturer`s instructions.

### Flow cytometry analysis

Staining of CAR T cells and tumor cells was performed as described previously (20). Briefly, cells were fixed in 1.5% PFA, followed by blocking in FACS Wash (FW) buffer (PBS + 1% BSA) for 20 min at room temperature. For surface staining, cells were directly stained in FW buffer. For intracellular staining, cells were pre-permeabilized in FWS buffer for 10 min, followed by 20 min staining in FWS using fluorescently labeled primary antibodies. Data were acquired on an LSRII flow cytometer (BD Biosciences) and analyzed with FlowJo software (version 10.1, BD Biosciences).

### Phenotypical characterization of CAR T cells

Eight days after activation, CAR T cells were either maintained alone or cocultured with SKBR3 tumor cells for 8 and 24 h under control and pro-oxidative conditions. Then, CAR T cells were collected, fixed, and stained to assess: i) CAR construct expression, ii) exhaustion state, and iii) memory state. The following antibodies were used for staining: i) CAR construct expression, IgG-PE (Southern Biotech, 2042-09); ii) exhaustion state CD3-PE (BD Biosciences, 345765), CD4-V450 (BD Biosciences Biosciences, 560345), CD8-FITC (BD Biosciences, 555366), PD1-APC (BioLegend, 367406), TIM3-APC-Cy7 (BioLegend, 345026), and LAG3-PE-Cy7 (BioLegend, 369310); and iii) memory state CD4-AF700 (BioLegend, 317426), CD8-APC-Cy7 (BioLegend, 344714), CD45RA-APC (BioLegend, 304112), CD45RO-PerCP-Cy5.5 (BioLegend, 304222), CCR7-PE-Cy7 (BioLegend, G043H7), CD62L-PE-Cy5 (BioLegend, 304808), Granzyme B-FITC (BD, 558132), IFN-γ-BV621 (BioLegend, 502536), CD127-BV421(BioLegend, 351310), and CD57-PE (BioLegend, 359612) according to the manufacturer’s instructions.

### nCounter mRNA analysis

CAR T cells were prepared and activated as described above. Eight days after activation, 1 × 10^6^ cells in each group were untreated or treated with 25 µM H_2_O_2_ for 1 h. Excess H_2_O_2_ was removed by centrifugation. Subsequently, cells were cultured alone or cocultured with SKBR3 tumor cells at a 5:1 T cell:tumor cell ratio for 6 h. Next, total RNA was isolated using RNA isolation kits (R2051, Zymogen). RNA yield and integrity were assessed using a NanoDrop spectrophotometer (Thermo Fisher Scientific) and Bioanalyzer (Agilent Technologies), respectively. Gene expression profiling was conducted using a NanoString nCounter Immune Exhaustion Pathway panel. Data processing and analysis were performed as previously described (21). mRNA code counts in each sample were extracted using nSolver Analysis Software Version 4.0 (NanoString Technologies).

Significantly up- and down-regulated genes were fed into the Ingenuity Pathway Analysis (IPA) software to acquire unbiassed insight into regulated pathways (22). IPA regulation z-score was used to identify biological functions influenced by pro-oxidative conditions and antioxidant enhancement. The top 10 positive and top 10 negative z-scores are presented.

### Redoxosome analysis

siTRX1- or siControl-transfected PBTs were prepared and activated as described above. Three days after activation, samples were collected and used for TRX1 trapping. The TRX1 trapping approach was based on the finding that mutated thiol-dependent oxidoreductases lacking the C-terminal cysteine of the CxxC motif (CxxS mutants) form long-lived mixed disulfide intermediates with target proteins. Thus, the target proteins remain covalently linked to the mutant oxidoreductase, which can be immunoprecipitated. After kinetic trapping, proteins that were oxidized and bound to mutated TRX1 (23) were analyzed by mass spectrometry. For trapping reactions, 1 × 10^7^ activated PBTs were used for each sample. Trapping was performed as described previously (17).

### LC-MS analysis and database search

Immunoprecipitation-enriched protein samples were separated by SDS-polyacrylamide electrophoresis. Each lane was cut in six pieces and processed as described previously (24). In brief, trypsin digestion was conducted overnight at 37°C. Reactions were quenched by addition of 20 µL 0.1% trifluoroacetic acid (TFA; Biosolve, Valkenswaard, The Netherlands), and supernatants were dried in a vacuum concentrator before LC-MS analysis. Nanoflow LC-MS^2^ analysis was performed using an Ultimate 3000 liquid chromatography system coupled to an QExactive HF mass spectrometer (Thermo Fisher Scientific). Samples were dissolved in 0.1% TFA, injected into a self-packed analytical column (75 µm x 200 mm; ReproSil Pur 120 C18-AQ; Dr Maisch GmbH), and eluted with a flow rate of 300 nl/min in an acetonitrile-gradient (3%–40%). The mass spectrometer was operated in data-dependent acquisition mode, automatically switching between MS and MS^2^. Collision-induced dissociation MS^2^ spectra were generated for up to 20 precursors with normalized collision energy of 29%.

Raw files were processed using MaxQuant version 1.6.12.0 (J. Cox, M. Mann, Nat Biotechnol 2008, 26, 1367) for peptide identification and quantification. MS^2^ spectra were searched against the Uniprot human proteome database (UP000005640_9606.fasta, downloaded Nov 2019) and the contaminants database provided with the MaxQuant software and using the Andromeda search engine with the following parameters: carbamidomethylation of cysteine residues as fixed modification; acetyl (Protein N-term), oxidation (M), and deamidation (Q, N) as variable modifications; and trypsin/P as the proteolytic enzyme, with up to two missed cleavages allowed. The maximum false discovery rate for proteins and peptides was 0.01, and a minimum peptide length of 7 amino acids was required. All other parameters were the default MaxQuant parameters. For quantitative analysis, intensity-based absolute quantification (iBAQ) was performed as previously described (25); this MaxQuant software algorithm normalizes the sum of all peptide intensities to the size of the protein to generate an iBAQ value for the protein that correlates with its absolute amount.

### Statistics

Statistical analyses were performed using GraphPad Prism 9.0 (GraphPad). Differences between two groups were evaluated by unpaired/paired two-tailed t-tests (as indicated in the figure legends), and differences among more than two groups evaluated by two-way analysis of variance. All data are presented as mean ± standard error of the mean (SEM). Statistical significance was determined by the *p*-values generated by statistical tests, with levels deemed significant, as follows: *p < 0.05, **p < 0.01, ***p < 0.001, and ****p < 0.0001.

## Results

### TRX1 is pivotal for redox homeostasis and T cell proliferation

**Figure 1:**
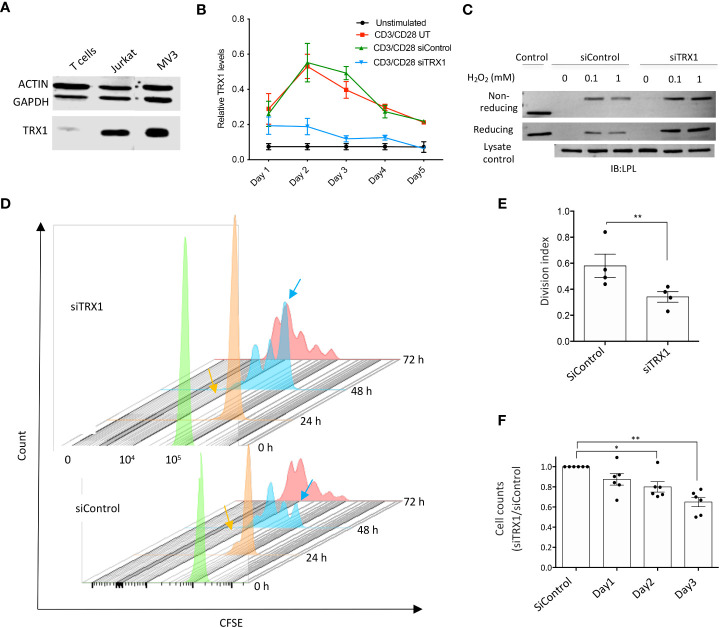
TRX1 is pivotal for redox homeostasis and T cell proliferation. **(A)** Representative immunoblot showing TRX1 levels in PBTs, MV3 cells, and Jurkat lymphoma cells. Lysates (20 µg) were loaded in each lane. Actin and GAPDH were used as loading controls (n = 3). **(B)** Kinetics of TRX1 expression in PBTs. PBTs were nucleofected with control or TRX1-specific siRNA and activated with plate-bound anti-CD3/anti-CD28 antibodies for up to 5 days. TRX1 expression was assessed by flow cytometry (n = 4). **(C)** Representative immunoblots showing TRX1-trapping oxidized LPL in peripheral blood T cells (PBTs). PBTs nucleofected with control or siTRX1-specific siRNA were treated with the indicated concentrations of H_2_O_2_ 3 days after activation, and trapping was performed (n = 3). **(D)** Proliferation of untransformed human PBTs over 3 days. Cell proliferation is indicated by diminished intensity of CFSE fluorescence per cell. Mean fluorescence intensity of CFSE on: 0 h (green), 24 h (orange), 48 h (light blue), and 72 h (red) is shown. Orange and light blue arrows indicate once-divided and undivided PBT populations at 24 and 48 h in the siControl and siTRX1 groups, respectively. **(E)** Division indices of control (siControl) or TRX1-specific (siTRX1) siRNA-transfected PBTs after 72 h. **(F)** Ratio of cell counts in siTRX1 and siControl transfected cells over 3 days. Data represent mean ± SEM (n ≥ 3; *p < 0.05, **p < 0.01).

We first measured TRX1 levels in T cells and tumor cell lines. TRX1 levels were markedly lower in peripheral blood T cells (PBTs) compared to tumor cells (**Figure 1A**). We hypothesized that low TRX1 levels in PBTs relative to tumor cells may contribute to their high sensitivity to ROS. To understand the role of TRX1 in T cells, PBTs were nucleofected with control (siControl) or TRX1-specific (siTRX1) siRNA. Activation of PBTs using plate-bound anti-CD3/anti-CD28 antibodies induced TRX1 expression, which peaked after 48 h and then gradually returned to baseline over 5 days (**Figure 1B**). Upregulation of TRX1 upon CD3/CD28 costimulation was almost completely abolished by TRX1-specific siRNA both at the peak (48 h) and at 72 h.

We have shown earlier that lowering TRX1 levels in tumor cells led to oxidation of the actin-bundling protein LPL (17). Since LPL plays an important role in the formation of the immune synapse, T cell activation and tumor cell killing (18), we next examined the correlation between TRX1 levels and LPL oxidation in PBTs. PBTs were treated with different H_2_O_2_ concentrations, and TRX1 trapping experiments were performed as previously described (17). TRX1 knockdown indeed correlated with elevated LPL oxidation in activated PBTs (**Figure 1C**). Consistently, we have observed elevated ROS levels in TRX1 knock-down cells using CM-H_2_DCFDA ROS sensor (data not shown).

To further elaborate TRX1’s function in T cells, proliferation of activated PBTs transfected with siControl or siTRX1 was monitored by a CFSE dilution assay. PBTs lacking TRX1 exhibited diminished proliferation (**Figures 1D, E**). Accordingly, T cell expansion post-activation was dampened in TRX1 knockdown cells (**Figure 1F**). These data underscore the importance of the TRX1-antioxidant system for T cell redox homeostasis and expansion capacity.

### Generation and characterization of antioxidant-enhanced CAR T cells

In light of our findings on the importance of TRX1 in T cells and published data on the hypo-responsiveness of T cells under pro-oxidative conditions (15, 16), we aimed to improve T cell resistance to the pro-oxidative solid TME and thereby increase their efficacy against and longevity in solid tumors. HER2-specific CAR T cells (HER2-CAR T cells) were selected as model system.

We generated and characterized CAR T cells stably expressing two antioxidant systems: TRX1, and GRX1.). These antioxidant genes were overexpressed under the control of the human phosphoglycerate kinase promoter, together with a HER2-specific CAR construct (**Supplementary Figure S1A**). HER2-CAR T cells or HER2-CAR T cells overexpressing GRX1 and TRX1 had comparable levels of HER2-CAR construct expression (**Supplementary Figure S1B**).

To monitor antioxidant gene expression after CAR T cell activation, CAR T cells expressing eGFP were also generated (**Supplementary Figure S1C**). As a marker of antioxidant expression, eGFP expression was comparable to CAR construct expression. When CAR T cells were lysed and immunoblotted, overexpression of TRX1 and GRX1 was clearly detected in TRX1-expressing and GRX1-expressing HER2-CAR T cells, respectively (**Supplementary Figures S1D–F**).

The influence of antioxidant enhancement on CAR T cell expansion capacity was also evaluated. Cell numbers 8 days after activation were comparable, although a trend toward higher expansion capacity was observed in TRX1-expressing HER2-CAR T cells (**Supplementary Figures S1G, H**).

### TRX1-expressing CAR T cells retain their capacity to form cytolytic synapses under pro-oxidative conditions

**Figure 2:**
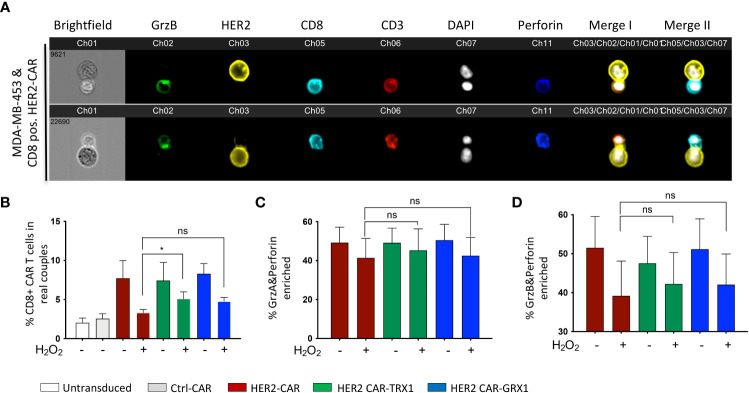
TRX1-expressing CAR T cells retain their capacity to form cytolytic synapses under pro-oxidative conditions. **(A)** Representative images of cytolytic immune synapses between CD8+ HER2-CAR T cells and MDA-MB-453 tumor cells. CAR T cells were untreated or pretreated with H_2_O_2_ for 30 min, then cocultured with MDA-MB-453 (1:1 ratio, 15 min). Cells were fixed and stained for granzyme B (green), HER2 (yellow), CD8 (cyan), CD3 (red), nuclei (DAPI, white), and perforin (blue). Merged images of CD3, HER2 and DAPI (merge I) and CD8, HER2 and DAPI (Merge II) are shown. **(B)** % CD8+ CAR T cells in synapses with MDA-MB-453 tumor cells. Data represent mean ± SEM (n ≥ 3; *p < 0.05, ns = non-significant). **(C, D)** Quantification of enrichment of **(C)** granzyme A and perforin, and **(D)** granzyme B and perforin at the immune synapse. Data represent mean ± SEM (n ≥ 3; *p < 0.05).

A fundamental step in T cell cytotoxicity toward tumor cells is recognition of tumor antigens and formation of a specific interaction zone, the cytolytic immune synapse, between T cells and tumor cells. To assess the influence of antioxidant protein expression on CAR T cells, the formation of cytolytic immune synapses between CD8+ CAR T cells and HER2-expressing MDA-MB-453 tumor cells was analyzed by inflow microscopy of > 25,000 cells (**Figures 2A-D**). **Supplementary Figure S2** shows the gating strategy, and **Supplementary Figure S3** shows the results for CD4+ CAR T cells. Analysis of immune synapses revealed that CD8+ HER2-CAR, HER2-CAR-TRX1, and HER2-CAR-GRX1 cells all formed significantly more cytolytic immune synapses with tumor cells than Ctrl-CAR and control T cells (untransduced, activated) (**Figure 2B**). Under pro-oxidative conditions mimicked by administration of exogenous H_2_O_2_ for 1 h, CAR T cells formed substantially fewer cytolytic immune synapses, while TRX1-expressing HER2-CAR T cells still formed significantly more synapses (**Figure 2B**).

Efficient tumor cell killing also involves translocation of granzymes A and B and perforin into immune synapses. Our analysis revealed a diminished proportion of CAR T cells with granzyme A, granzyme B, and perforin enrichment into the interaction zone under pro-oxidative conditions by trend (**Figures 2C, D**). And antioxidant enhancement had no significant impact on this outcome.

Tumor cell recognition by CD4+ T cells is also fundamental to tumor cell elimination by direct release of the proinflammatory cytokines interferon (IFN)-γ and tumor necrosis factor (TNF)-α, and by modulating the activity of other immune cells including CD8+ CAR T cells. Further, CD4+ T cells also exert direct cytotoxicity through granzyme B and perforin release during viral infections (reviewed in (26)). Thus, CD4+ CAR T cell synapse formation capacity was also investigated. Similar to CD8+ cytotoxic CAR T cells, CD4+ CAR T cells formed comparable numbers of conjugates with MDA-MB-453 tumor cells (**Supplementary Figure S3A**). The number of synapses was significantly diminished when CD4+ CAR T cells were pre-treated with H_2_O_2_, while similar to CD8+ CAR T cells (**Figure 2B**), the CD4+ CAR T cells expressing TRX1 or GRX1 significantly retained their synapse formation capacity under pro-oxidative conditions. CD4+ CAR T cells had low levels of granzyme A, granzyme B, and perforin expression that were not altered by ROS treatment (**Supplementary Figures S3B, C**).

Overall, TRX1 expression rescued ROS-induced reduction of CAR T cell immune synapse formation capacity.

### CAR T cells efficiently kill target tumor cells over time

To determine the optimal timepoint to assess CAR T cell cytotoxicity to tumor cells, the kinetics of tumor cell killing by CAR T cells were assessed using time-lapse imaging. CAR T cell cytolytic granules were stained using Lysotracker red and SKBR3 tumor cells were stained using caspase 3/7 sensor to detect tumor cell apoptotic signals followed by time-lapse microscopy for 24 h (**Supplementary Figures S4A, B**). After 24 h coculture, most tumor cells were killed, and CAR T cells formed clusters (**Supplementary Figure S3A**). CAR T cells started to interact with tumor cells after 30 min and the Lysotracker red signal (red arrows) was detectable in tumor cells as early as 4 h later. Tumor cell apoptotic signals (marked in green and with green arrows) were detectable 8 h after coculture initiation (**Supplementary Figure S3B**). Several HER2-CAR T cells interacted with one tumor cell. In most interactions, cytolytic granule release into the interaction zone was detected as an increase in Lysotracker red signal (**Supplementary Figure S3B**, red arrows). The final signs of tumor cell killing (cell shrinkage and lysis) were observed after 18 h of coculture and cleared after 22 h. These results clearly demonstrate time-dependent killing of tumor cells by serial interactions with a number of CAR T cells.

### Cytotoxicity of HER2-CAR T cells, GRX1- and TRX1-enhanced HER2-CAR T cells under pro-oxidative conditions

**Figure 3:**
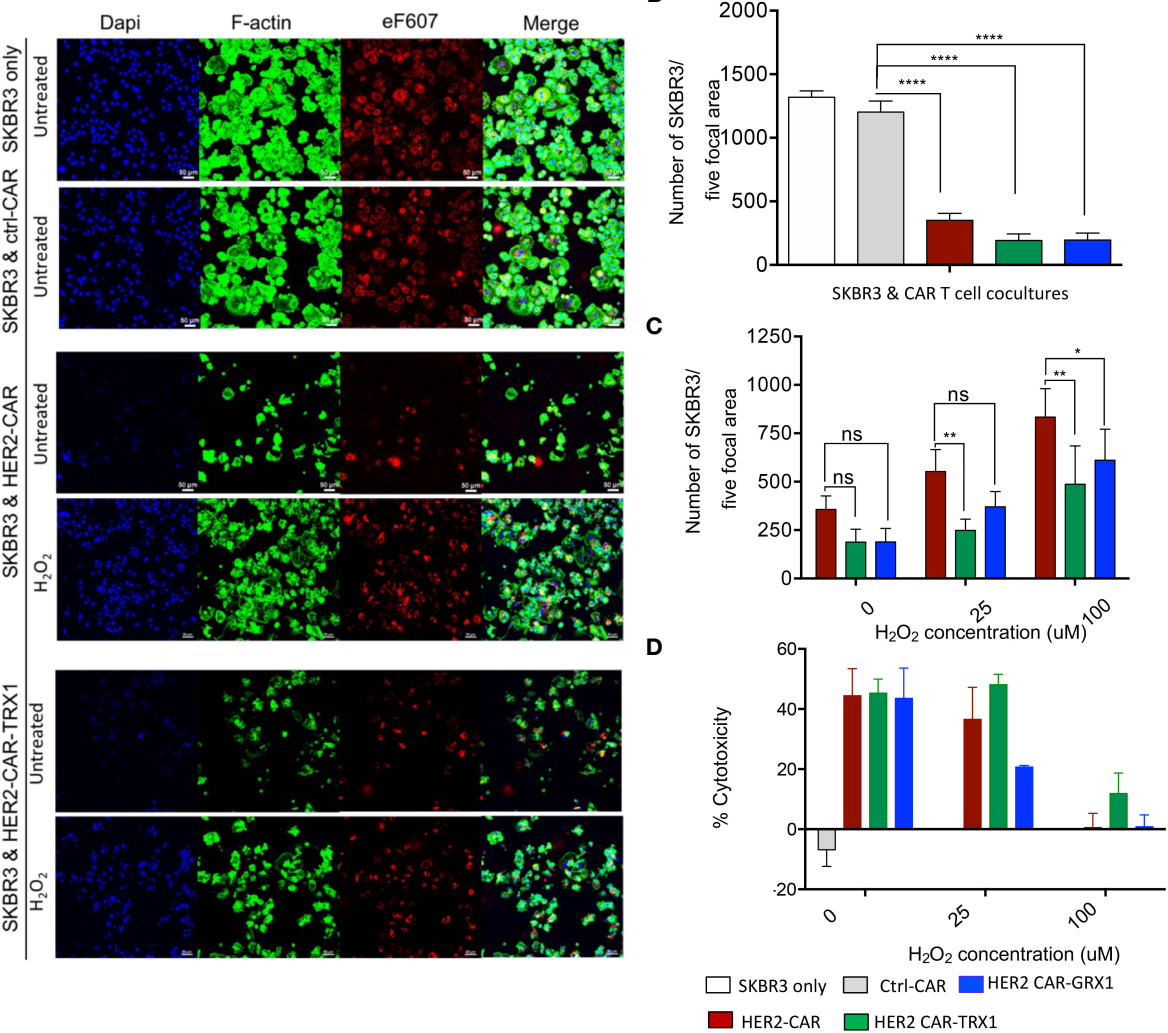
Cytotoxicity of HER2-CAR T cells and Grx1-, and TRX1-enhanced HER2-CAR T cells under pro-oxidative conditions. **(A)** SKBR3 tumor cells were pre-stained with eF607 (red) and seeded on coverslips. The next day, ctrl-CAR T cells, HER2-CAR T cells, or antioxidant-enhanced HER2-CAR T cells were added onto the coverslips and cocultured for 24 h. Then, cells were stained for nuclei (DAPI, blue), and F-actin (phalloidin, green). Large scan (3 x 3 *xy*) images were acquired using a 20x objective and cells from five focal planes quantified (n = 3). **(B)** Quantification of SKBR3 cells in five focal planes. Percentage of SKBR3 cells in cocultures were calculated by taking the ratio of the number of SKBR3 cells in coculture to that of SKBR3 cells under control (SKBR3 only) conditions (n = 4). **(C)** Quantification of SKBR3 cells in five focal planes (n = 4). After coincubation (24 h) of untreated or H_2_O_2_-pre-treated HER2-CAR, HER2-CAR-TRX1, and HER2-CAR-GRX1 T cells in a 5:1 ratio with SKBR3 cells, a microscopy-based cytotoxicity assay was performed. Data represent mean ± SEM (n ≥ 3; *p < 0.05, **p < 0.01). **(D)** LDH release assay showing percentages of CAR T cell cytotoxicity to tumor cells at the indicated CAR T cell:tumor cell ratios. Untreated or H_2_O_2_-pre-treated CAR T cells and SKBR3 cells were coincubated at the indicated ratios for 24 h. LDH activity in supernatants was determined by measuring spectrophotometric absorbance at 490 nm (n = 3). Data represent mean ± SEM (n ≥ 3; *p < 0.05, **p < 0.01, **** p < 0.00001, ns = non-significant).

To evaluate whether the expression of antioxidants in HER2-CAR T cells influences their cytotoxic capacity in an immunosuppressive environment due to pro-oxidative conditions, we established a microscopy-based cytotoxicity assay (**Figures 3A-C** and **Supplementary Figure S5A–D**). Lactate dehydrogenase (LDH) release assays were also used to investigate HER-CAR T cell cytotoxic capacity (**Figure 3D** and **Supplementary Figure S5E**).

Briefly, SKBR3 tumor cells were pre-stained with eF607 dye and allowed to adhere on coverslips in cell culture plates overnight. The next day, ctrl-CAR, HER2-CAR, or antioxidant-enhanced HER2-CAR T cells were added to the tumor cells at the indicated ratios and co-incubated for 8 or 24 h. Then samples were fixed, immunostained, and imaged by confocal microscopy (**Figure 3A**). Death of tumor cells leads to the detachment of the cells from the plate. To quantify CAR T cell induced tumor cell killing, cells per focal plane were counted and calculated using automated region of interest (ROI) analysis, based on DAPI and eF607 staining (**Supplementary Figures S5A–D**). The microscopy-based cytotoxicity assays clearly revealed killing of SKBR3 tumor cells by HER2-CAR T cells and by antioxidant-enhanced HER2-CAR T cells, but not by ctrl-CAR T cells (**Figure 3B**).

Next, we evaluated whether TRX1 or GRX1 expression in HER2-CAR T cells would protect them against the immunosuppressive effects of a pro-oxidative environment. To mimic a pro-oxidative environment, CAR T cells were pretreated with sublethal concentrations of H_2_O_2_ (25 or 100 µM) for 30 min prior to coincubation with HER2-expressing tumor cells; these H_2_O_2_ concentrations are above physiological ROS levels; however, higher concentrations of H_2_O_2_ are necessary to bypass cellular antioxidant capacity and induce sufficient global protein oxidation levels and are commonly used in redox studies (27, 28).

HER2-CAR T cell cytotoxicity was reduced in a dose-dependent manner in a pro-oxidative micromilieu (**Figures 3C, D**), while TRX1- and GRX1-expressing HER2-CAR T cells retained a higher level of cytotoxicity, even under high H_2_O_2_ conditions (100 µM H_2_O_2_) (**Figure 3C**). The cytotoxicity assay based on LDH release confirmed that particularly HER2-CAR-TRX1 T cells retained their killing capacity in a pro-oxidative environment (**Figure 3D**). Hence, CAR T cells can be empowered to overcome a pro-oxidative environment by co-expression of TRX1 and, to a lesser extent, GRX1.

### Release of effector cytokines under pro-oxidative conditions

**Figure 4:**
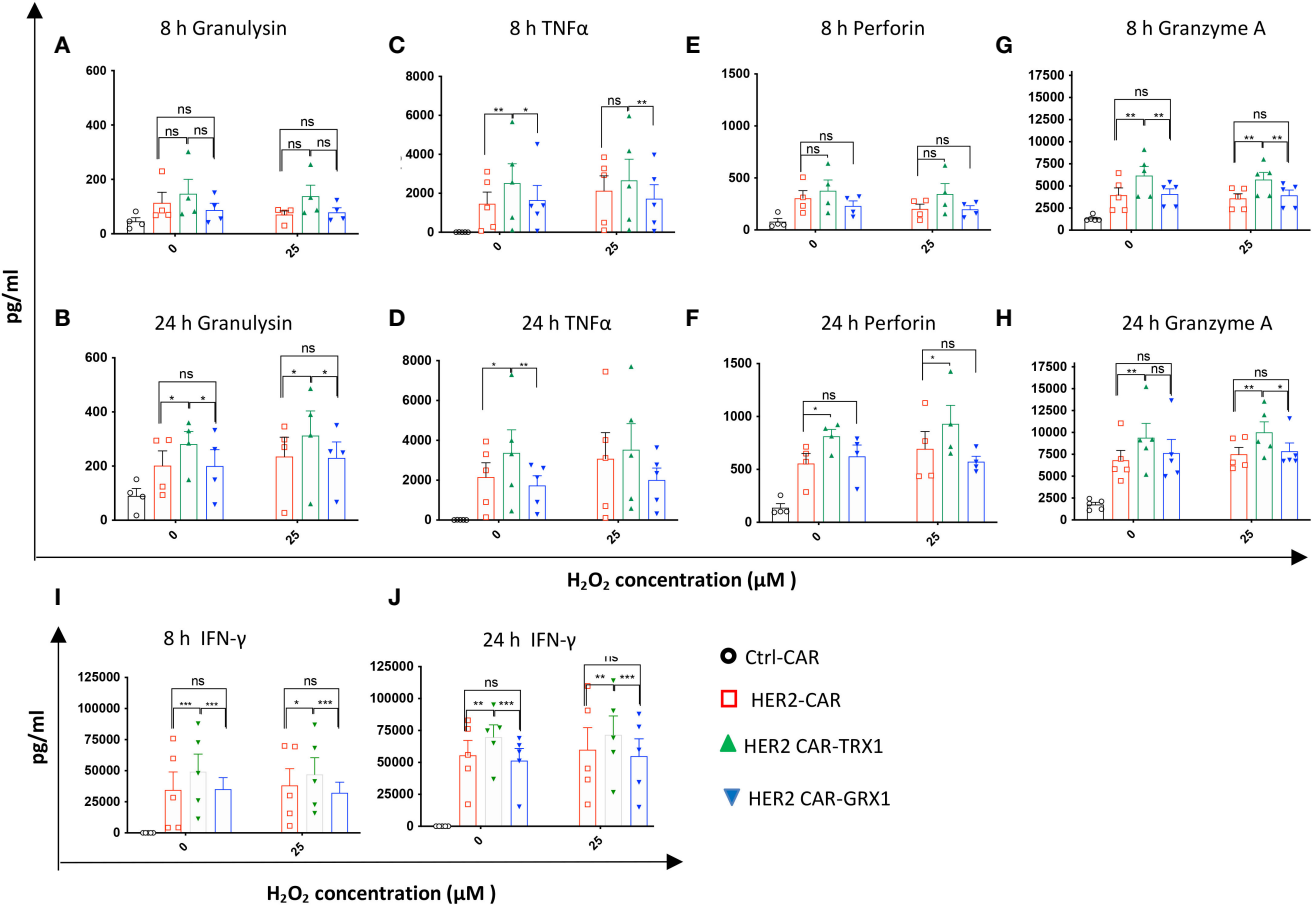
Release of effector cytokines under pro-oxidative conditions. After coculture of CAR T cells with SKBR3 cells (8 and 24 h), supernatants were collected and evaluated using the human CD8/NK panel 13-plex **(A–H)** and IFN-γ ELISA assay **(I, J)**. Cytokines differentially released by HER2-CAR T and HER2-CAR-TRX1 T cells are shown. Release of granulysin after **(A)** 8 h, **(B)** 24 h; TNFα after **(C)** 8 h, **(D)** 24 h; Perforin after **(E)** 8 h, **(F)** 24 h; and granzyme A after **(G)** 8 h, **(H)** 24 h. **(I, J)** Release of IFN-γ after **(I)** 8 and **(J)** 24 h coculture. Data represent mean ± SEM (n ≥ 3; *p < 0.05, **p < 0.01, *** p < 0.001, ns = non-significant

To further characterize the cytotoxic activity of antioxidant-enhanced CAR T cells, the release of effector cytokines was analyzed in the supernatants of SKBR3 tumor cells co-cultured with HER2-CAR T cells or antioxidant-enhanced HER2-CAR T cells. First, comparison of cytokine levels in supernatants of HER2-CAR T cells cultured alone or with SKBR3 clearly indicated upregulation of most analyzed cytokines including interleukin (IL)-2 (not shown), granzyme A, granulysin, perforin, IFN-γ, and TNF-α under coculture conditions (**Supplementary Figure S6**), indicating antigen recognition and activation of HER2-CAR T cells directed against HER2+ SKBR3 cells. Then we analyzed cytokines in supernatants collected from cocultures of HER2-CAR T cells or antioxidant-enhanced HER2-CAR T cells and SKBR3 cells. Among the 13 effector cytokines evaluated, levels of five (granulysin, TNF-α, granzyme A, perforin, and IFN-γ) differed significantly between HER2-CAR and HER2 CAR-TRX1 T cells (**Figure 4**) Of these, granulysin release differed significantly under both control and pro-oxidative conditions at 24 h, but not at 8 h (**Figures 4A, B**), while TNF-α release differed significantly under control conditions at 8 h and 24 h (**Figures 4C, D**). Perforin release was significantly higher in TRX1-expressing CAR T cells compared to control CAR T cells only after 24 h both under control and pro-oxidative conditions. Furthermore, Granzyme A release was significantly higher in TRX1-expressing CAR T cells at 8 h and 24 h after coculture under control and pro-oxidative conditions. Interestingly, GRX1-expressing HER2 CAR T cells and control HER2 CAR T cells did not show a difference in cytokine release under both control and pro-oxidative conditions (**Figures 4E-H**). Further, TRX1-expressing CAR T cells released elevated levels of IFN-γ under both control and pro- oxidative conditions, relative to HER2-CAR T cells (**Figures 4I, J**). After 8 and 24 h coculture, IFN-γ levels in supernatants from HER2-CAR-TRX1 T cells were 2-fold and 1.5-fold higher, respectively, than in HER2-CAR T cell cultures. These data provide clear evidence that TRX1-expressing HER2-CAR T cells release more effector cytokines under pro-oxidative conditions than HER2-CAR T cells and are superior to GRX1-expressing HER2-CAR T cells.

### TRX1 and GRX1 overexpression does not influence tumor-cell induced CAR T cell exhaustion

**Figure 5:**
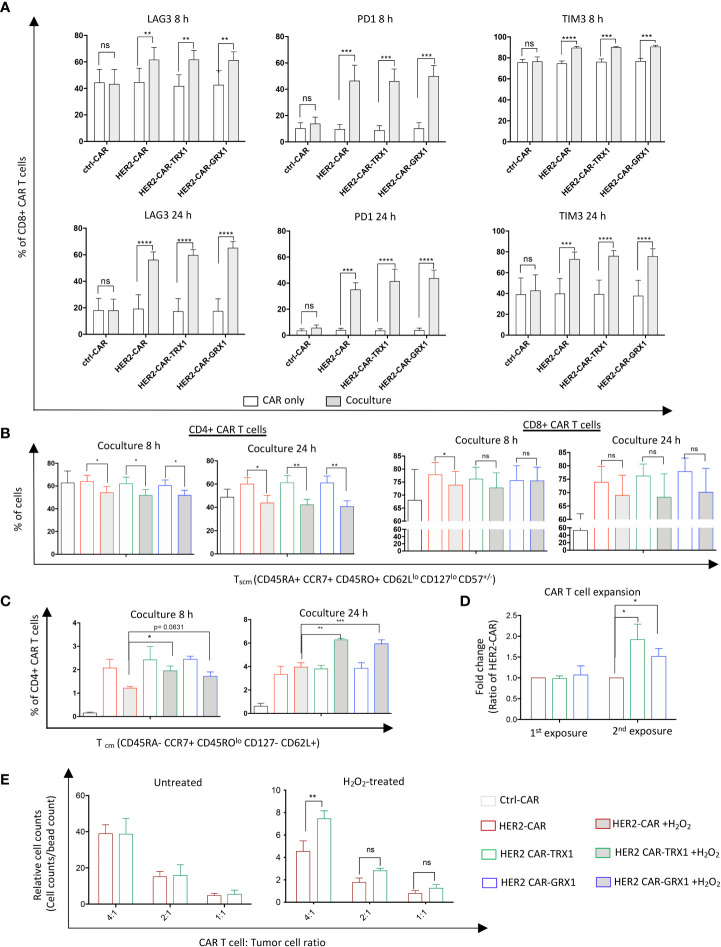
TRX1 and GRX1 overexpression does not influence tumor cell-induced CAR T cell exhaustion. Ctrl-, HER2-, or antioxidant-enhanced CAR T cells were untreated or treated with H_2_O_2_ and cocultured with SKBR3 cells for 8 and 24 h. Then, CAR T cells were collected and immunostained for exhaustion markers or markers to discriminate memory cell phenotypes. **(A)** Percent expression of LAG3 (left), PD1 (middle), and TIM3 (right) in CD8+ CAR T cells after 8 (upper graphs) and 24 (lower graphs) h of CAR only culture (white bars) and CAR T cell/SKBR3 coculture (grey bars). Data represent mean ± SEM (n ≥ 3; *p < 0.05, **p < 0.01, ***p < 0.001, ****p < 0.0001, ns = non-significant). **(B)** Percent CD4+ (left graphs) and CD8+ (right graphs) T_scm_ CAR T cells in 8 and 24 h CAR T cell/SKBR3 cocultures under control and pro-oxidative conditions. **(C)** Percent CD4+ CD45RO^lo^ T_cm_ cells at 8 h (left graph) and 24 h (right graph) CAR T cell/SKBR3 cocultures under control and pro-oxidative conditions. Data represent mean ± SEM (n ≥ 3; *p < 0.05, **p < 0.01, ***p < 0.001, ns = non-significant). **(D)** Expansion capacity of CAR T cells after single (1st exposure) and double (2nd exposure) culture with SKBR3 tumor cells. After coculture (5 days) CAR T cells were collected and stained with anti-CD3 antibody and viability dye. Then, samples were mixed with equal amounts of beads. Relative expansion was calculated in relation to bead counts by flow cytometry. Data represent mean ± SEM (n ≥ 3; *p < 0.05, ns = non-significant). **(E)** Expansion capacity of CAR T cells in SKBR3/CAR T cell cocultures after 5 days. Data represent mean ± SEM (n ≥ 3; *p < 0.05, **p < 0.01, ns = non-significant).

Next, to characterize the influence of antioxidant proteins on T cell exhaustion, CAR T cells were maintained untreated or treated with 25 µM H_2_O_2_ for 1 h. Then HER2-CAR and antioxidant-enhanced HER2-CAR T cells were cultured alone or with SKBR3 tumor cells for 8 and 24 h. Programmed death 1 (PD1), T-cell immunoglobulin and mucin-domain containing-3 (TIM3), and Lymphocyte-activation gene 3 (LAG3) expression levels on the cell surface were evaluated to assess T cell exhaustion state (gating strategy in **Supplementary Figure S7**). After 8 and 24 h coculture, expression of all three exhaustion markers was significantly upregulated in both CD8+ (**Figure 5A**) and CD4+ (**Supplementary Figure S9A**) HER2-, but not ctrl-, CAR T cells, consistent with the current view that tumor cell/T cell interactions in the TME induce expression of exhaustion markers in T cells (3). Further analysis of exhaustion marker levels in CD8+ and CD4+ T cells indicated that ROS significantly increased TIM3 expression but did not influence PD1 expression levels (**Supplementary Figure S9B**). Additionally, ROS caused a slight increase in LAG3 expression in HER2-CAR T cells and HER2-CAR T cells expressing GRX1 after 8 h coculture, but not in HER-CAR T cells expressing TRX1. These data indicate that TRX1 may prevent the ROS-induced elevation of the exhaustion marker LAG3, but does not appear to influence tumor cell-induced expression of PD1 or ROS-induced expression of TIM3.

### TRX1-expressing HER2-CAR T cells have slightly elevated levels of CD45RO^lo^ central memory cells under pro-oxidative conditions

Memory stem T cells (T_scm_) and central memory T cells (T_cm_) cells have superior survival and antitumor activity compared to effector memory T cells (T_em_) and effector T cells (T_eff_) cells (29, 30). Thus, we assessed the memory state of CAR T cells cultured alone or together with SKBR3 tumor cells (**Figures 5B, C**, and **Supplementary Figure S8**). CAR T cells were treated with H_2_O_2_ and cocultured as described above. To identify the memory populations, a newly-established memory panel was employed that can discriminate naïve T cells (T_na_), T_cm_, CD45RA+ effector memory T cells (T_emra_), T_scm_, T_eff_, and T_em_ (gating strategy in **Supplementary Figure S8**).

ROS significantly diminished the T_scm_ population in both CD4+ (left graphs) and CD8+ (right graphs) CAR T cells (**Figure 5B**), while increasing the proportion of T_em_ cells (**Supplementary Figure S9C**). Proportions of the main memory cell types were not influenced by overexpression of TRX1 or GRX1; however, a minor population termed CD45RO^lo^ central memory CD4+ T cells was significantly increased in TRX1-expressing HER2-CAR T cells relative to HER2-CAR T cells under pro-oxidative conditions at 8 h and 24 h. (**Figure 5C**).

### TRX1-expressing HER2-CAR T cells have enhanced expansion capacity in tumor cell cocultures

Antitumor T cells need to survive and repeatedly kill tumor cells in immunosuppressive, pro-oxidative environments. To mimic these conditions *in vitro*, we cocultured CAR T cells with SKBR3 tumor cells for 5 days. Then aliquots of the cells were collected, and relative CAR T cell counts were assessed by flow cytometry (**Figure 5D**). The remaining cells were further cocultured with freshly-seeded SKBR3 tumor cells (repeated exposure), and relative CAR T cell counts were calculated again 5 days after the second exposure. Antioxidant-enhanced CAR T cells clearly had superior expansion capacity (**Figure 5D**). Thus, higher antioxidant levels in HER2-CAR-TRX1 and HER2-CAR-GRX1 cells may provide direct protection to CAR T cells in this environment.

To validate this finding, HER2-CAR-TRX1 and HER2-CAR T cells were cocultured with SKBR3 tumor cells at different ratios under control and pro-oxidative conditions. Exogenous ROS administration strongly reduced relative counts of HER2-CAR T cells and TRX1-expressing HER2-CAR T cells 5 days after coculture (**Figure 5E**, compare relative counts in untreated (left graph) and H_2_O_2_-treated (right graph)). Consistently, under pro-oxidative conditions HER2-CAR-TRX1 T cells expanded more strongly than HER2-CAR T cells.

### TRX1 regulates the reduction of key proteins important for T cell antitumor functions

**Figure 6:**
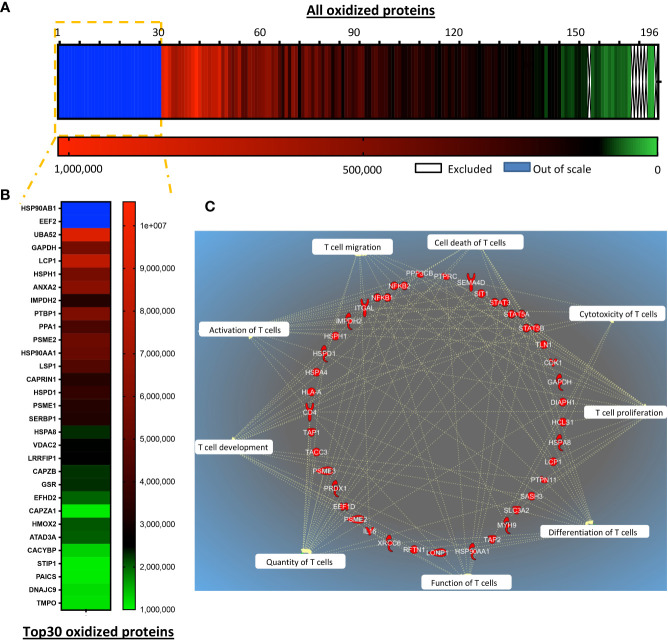
TRX1 regulates reduction of key proteins important for T cell antitumor functions. siControl- or siTRX1-transfected T cells were untreated or treated with H_2_O_2_ for 5 min. Then intracellular thiol reactions were immediately frozen by NEM alkylation and samples subjected to TRX1 kinetic trapping. TRX1-bound oxidized proteins were separated on SDS-polyacrylamide gels under reducing conditions and analyzed by mass spectrometry. **(A)** Heat map showing the oxidation state of 196 highly-oxidized proteins. **(B)** Top 30 highly-oxidized proteins. **(C)** Pie chart showing the top 10 functions associated with the oxidized proteins.

**Figure 7:**
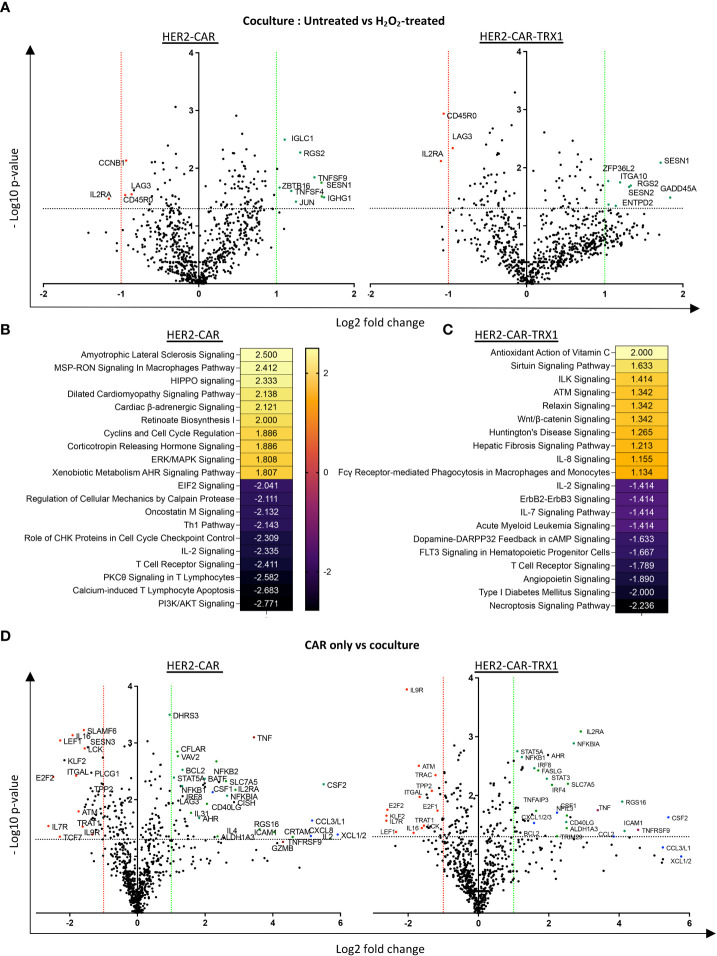
A pro-oxidative micromilieu alters CAR T cell gene expression. Ctrl-, HER2-, and TRX1-enhanced CAR T cells were untreated or treated with H_2_O_2_ for 1 h. Cells were then cultured alone or cocultured with SKBR3 cells at a 5:1 CAR T cell: SKBR3 ratio. Cells were harvested 5 h later, and total RNA isolated and processed by nCounter mRNA analysis. **(A)** Volcano plot showing Log_2_ transformed mRNA expression in cocultured HER2-CAR T cells (left) and HER2-CAR-TRX1 T cells under control vs pro-oxidative conditions. **(B, C)** Ingenuity pathway analysis (IPA) of differentially regulated genes in untreated vs H_2_O_2_-treated **(B)** HER2-CAR and **(C)** HER2-CAR-TRX1 T cells under coculture conditions. **(D)** Expression differences between CAR T cell only vs CAR T cell/SKBR3 cocultures of HER2-CAR T cells (left) and HER2-CAR-TRX1 T cells (right) under control conditions.

Post-translational regulation of protein functions through cysteine oxidation under pro-oxidative conditions can change protein function, localization, and interaction partners; this enzyme-independent posttranslational process results in rapid regulation of cellular responses. We hypothesized that several proteins may be oxidized in T cells with low antioxidant capacity, compromising their functions. To assess the molecular evidence for the improved function of TRX1-expressing CAR T cells, global protein oxidation was investigated by TRX1 kinetic trapping (17). After immunoprecipitation, oxidized proteins were eluted, and 196 proteins identified by mass spectrometry were quantified by intensity-based absolute quantification (IBAQ) (**Figures 6A, B**). IBAQ was used, rather than label-free quantitation intensity, because it allows protein sorting according to their absolute amounts. Importantly, the actin bundling protein LPL was among the top 30 oxidized proteins (17) (**Figure 6B**). Further, PTP1B, a redox-regulated protein tyrosine phosphatase involved in the cell cycle and cell proliferation (31), was also highly oxidized. Other highly oxidized proteins were heat shock family proteins (HSPH1, HSP90AB1, STIP1, HSPA8, and HSPD1) and nucleotide and protein biosynthesis-related proteins (IMPDH2, ALDH18A1) (**Figure 6B**). Intriguingly, glyceraldehyde 3-phosphate dehydrogenase (GAPDH) (**Figure 7B**) and four other enzymes of the glycolytic pathway were also highly oxidized (**Supplementary Table S1**).

Ingenuity pathway analysis (IPA) was performed to provide an unbiased understanding of T cell functions potentially influenced by global protein oxidation and of the protective roles of TRX1. IPA revealed that oxidized proteins were annotated to processes including cell proliferation, cell expansion, activation, cytotoxicity, and effector functions of T cells (**Figure 6C**).

### A pro-oxidative micromilieu alters CAR T cell gene expression

Various signaling molecules and transcription factors are regulated by ROS (reviewed in (12)), potentially influencing cell differentiation and activation, which are important for efficient antitumor responses. Therefore, the expression of several genes in HER2-CAR and HER2-CAR-TRX1 T cells was assessed under control or pro-oxidative conditions using the nCounter^®^ Immune Exhaustion Panel, allowing real-time quantification of 785 genes at the mRNA level.

Comparisons of mRNA counts in untreated versus H_2_O_2_-treated HER2-CAR and HER2-CAR-TRX1 T cells under coculture conditions revealed differing responses (**Figure 7A**). In particular, *CD45RO* expression was significantly lower in TRX1-expressing CAR T cells under pro-oxidative conditions, while it was slightly but significantly lower in HER2-CAR T cells under pro-oxidative conditions, consistent with our data presented in **Figure 5C**. Importantly, *IL2RA* levels were also significantly lower in both groups under pro-oxidative conditions.

IPA was performed to clarify the pathways regulated in HER2-CAR and HER2-CAR-TRX1 T cells under control and pro-oxidative conditions. Genes with significant differences in expression were fed into the software, and the z-scores of the top 10 upregulated and downregulated pathways selected (**Figures 7B, C**). Intriguingly, phosphoinositide 3-kinase (PI3K)/AKT signaling, PKC-θ T cell receptor signaling, and IL-2 signaling were among the top downregulated pathways in HER2-CAR T cells under pro-oxidative conditions. In contrast, these pathways were less influenced under pro-oxidative conditions in TRX1-expressing HER2-CAR T cells and were not detected in the top10 downregulated pathways.

The upregulated pathways in both groups were mostly associated with stress responses (**Figures 7B, C**), but antioxidant signaling (Antioxidant action of vitamin C) was the top positively-regulated pathway only in TRX1-expressing CAR T cells, but not in HER2 CAR T cells (**Figure 7C**).

Furthermore, gene expression related to proteins required for T cell activation and antitumor function was significantly upregulated only when T cells were cocultured with tumor cells (**Figure 7D** and **Supplementary Figure S10A**). One key gene, *TNFRSF9* encoding CD137, was strongly upregulated on engagement with tumors; however, it was only significantly upregulated in TRX1-expressing CAR T cells but not HER2-CAR T cells. Since CD137 is a potent tumor-specific T cell marker (32), this finding may provide another explanation for the superior antitumor functions of TRX1-expressing CAR T cells. Other important regulated proteins included TNF-family proteins and transcription factors (encoded by *STAT3A, STAT5A, NF-KBI, NF-KBII, IL2RA*, and *CD40LG*) (**Figure 7D**, green dots). Overexpression of TNF family proteins (red dots) was prominent in in both HER2-CAR and HER2-CAR-TRX1 T cells. Similarly, strong and significant increases in various chemokine receptors were observed in both HER2-CAR and HER2-CAR-TRX1 T cells cocultured with tumor cells (**Figure 7D**, blue dots). This finding was confirmed by comparison of positively or negatively regulated pathways in CAR T cells cultured alone under control and pro-oxidative conditions (**Supplementary Figure S10B, C**).

Tumor cell coculture reduced expression of the homeostatic cytokine receptor *IL7R* in both HER2-CAR and HER2-CAR TRX1 T cells. Interestingly, expression of TCF7, a predictive marker for successful immunotherapy and patient survival (33, 34), was only significantly and strongly downregulated in HER2-CAR T cells, but not in TRX1-expressing HER2-CAR T cells, when cocultured with tumor cells.

Together, gene expression analysis of CAR T cells on coculture with tumor cells under control or pro-oxidative conditions revealed multiple molecular explanations for the improved functions of TRX1-expressing CAR T cells under pro-oxidative conditions.

## Discussion

In solid tumors, tumor cells themselves, hijacked immune cells, and nonimmune cells produce ROS chronically, representing a key immunosuppressive factor in the TME (3, 35). While tumor cells have developed strategies to cope with high ROS levels by upregulating their antioxidant systems (36, 37), immune cells, particularly T cells, have very low antioxidant capacity. Hence, hyporesponsive antitumor T cell function in solid tumors can be associated with the pro-oxidative tumor microenvironment. In this study, we investigated the molecular and functional consequences of enhancing the antioxidant capacity of CAR T cells by overexpressing antioxidant proteins. Our findings provide clear molecular and functional evidence that TRX1-enhancement can help CAR T cells to overcome a pro-oxidative micromilieu.

Our findings are in line with a recent *in vitro* study showing that overexpression of CAT, which catalyzes the decomposition of H_2_O_2_ to water and oxygen, protects HER2-CAR T cells from exogenously administered ROS (19). Furthermore, since CAT nonspecifically acts on H_2_O_2,_ we prioritized the use of antioxidants with a higher substrate specificity (TRX1 and GRX1). Expression of TRX-1 turned out to be most effective to enhance the resistance of CAR T cells towards a pro-oxidative TME. In this context, we found that perforin and granzyme A release were significantly higher after 24 h in TRX1- but not GRX1-expressing HER2 CAR T cells under both control and pro-oxidative conditions. In support of this differential role, GRX could not compensate for TRXR1 loss in T cells (10), despite the synergistic contribution of GRX1 and TRX1 to redox equilibrium in other cell types (38–41).

The importance of TRX1 for T cell functions is further supported by experiments performed with knock-out mice that have shown that thioredoxin reductase (TRXR1), which is important to keep TRX1 active, is essential for fueling DNA synthesis during T cell metabolic reprogramming (10, 42). The direct influence of TRX-1 on gene expression levels or global redox homeostasis of NK cells or T cells has so far not been investigated. Therefore, our study - to our knowledge - provides the first comprehensive mechanistical insight into the superior T cell functionality gained upon expression of TRX1 in human (CAR) T cells.

To successfully eradicate tumors, tumor-specific T cells need not only to survive, proliferate, and migrate, but also to perform cytolytic functions, and generate long-lasting immune memory against the tumor, eventually leading to tumor regression. Antioxidant enhancement, particularly with TRX1, enhances the expansion capacity of CAR T cells and their cytotoxic functions. In this context, the retention of cytolytic immune synapse formation and effector cytokine release capacity, as well as cytotoxicity due to TRX1 enhancement, provides strong evidence for the importance of antioxidant empowerment for successful solid tumor immunotherapy.

A long-lasting immune response in the solid tumor environment requires superior memory cell phenotypes without CAR T cell exhaustion. Here, we found that the T_scm_ population was downregulated when CAR T cells were pre-treated with ROS before coculture with tumor cells. By contrast, T_em_ cells were significantly upregulated under pro-oxidative settings. Hence, a pro-oxidative tumor micromilieu can influence longevity and antitumor functions by changing antitumor T cell memory phenotypes. Importantly, antioxidant enhancement by TRX1 expression significantly increased the CD45RO^lo^ central memory cell population under pro-oxidative conditions, providing evidence that this approach may assist in partial retention of superior antitumor T cell memory phenotypes.

T cells in the TME are persistently exposed to tumor antigens and to inflammatory conditions that induce a dysfunctional state termed exhaustion (43) that is characterized by high levels of immunoinhibitory molecules and poor effector functions. Among exhaustion markers, TIM3, LAG3, and PD1 are highly expressed on T cells in several cancer types and their triple knockdown can enhance CAR T cell antitumor functions (44). We observed strong induction of all three exhaustion markers in T cells cocultured with tumor cells. ROS slightly enhanced LAG3 expression and strongly induced TIM3, without influencing PD1 levels on the surface of HER2-CAR T cells; however, no ROS-induced increase in LAG3 expression was observed in TRX1-expressing HER2-CAR T cells. Together, these data indicate that tumor cell- or ROS-induced T cell exhaustion must be addressed using strategies in addition to an antioxidant enhancement. One potential strategy would be the use of intrinsically modified CAR T cells that lack the exhaustion molecules (44, 45).

ROS can alter cell behavior by mediating post-translational thiol oxidation modifications and altering the expression of several proteins; the latter can also be indirectly regulated by oxidation of key molecules such as transcription factors. Functional annotation of oxidized proteins with T cell antitumor functions provided a molecular explanation for the loss of T cell functions in a pro-oxidative micromilieu. These results of this study represent the first comprehensive redoxosome analysis in T cells. Importantly, TRX1, as a regulator of the redox state of highly oxidizable proteins, seems to counter-balance the negative influence of exogenous ROS on antitumor functions of T cells.

Transcriptomic analysis of specific genes involved in immune activation, suppression, immune checkpoint receptors, and metabolic genes using the nCounter panel generated molecular evidence for tumor cell-induced downregulation of antitumor functions and for the beneficial effects of TRX1 enhancement. First, the comparison of gene expression in CAR T cells, either untreated or pre-treated with H_2_O_2_, under coculture conditions revealed that *IL2RA* was strongly downregulated, providing an explanation for the reduced proliferative capacity of CAR T cells under pro-oxidative conditions.

Furthermore, analysis of gene expression in CAR T cells cultured alone or co-cultured with tumor cells revealed marked differences between HER2-CAR and HER2-CAR-TRX1 T cells. Specifically, the reduction of *IL7R* levels under pro-oxidative conditions could explain the observed reductions in T_scm_ and T_cm_ populations, as IL-7 induces generation of these T cell populations (46); however, antioxidant enhancement did not influence *IL7R* levels under pro-oxidative conditions, suggesting that additional strategies to empower CAR T cells should be considered.

Downregulation of *TCF7* expression following tumor cell coculture was observed in HER2-CAR, but not HER2-CAR-TRX1, T cells, which could be another explanation for the improved functionality of HER2-CAR T cells expressing TRX1. The transcription factor TCF1 encoded by *TCF7* is critical for T cell development and differentiation. In cancer immunotherapy, exhausted T cells expressing TCF1 possess stem-like qualities, relative to exhausted T cells not expressing TCF1 (47) and have improved self-renewal capacity and functionality (48). TCF1 expression is also positively correlated with favorable prognosis in patients with melanoma after checkpoint blockade therapy (33). Our data highlight that TRX1-enhancement can help preserve TCF1 expression in CAR T cells and thereby preserve CD8+ T cell function under pro-oxidative conditions.

Considering the low tumor infiltration, low efficacy, and low expansion capacity of T cells in the majority of pro-oxidative solid tumors, our results provide a comprehensive molecular explanation for the failure of T cell antitumor responses and for the protective role of TRX1 under pro-oxidative conditions. The solid tumor TME poses several challenges to tumor-fighting T cells, resulting in low efficacy of T cell-based therapies against solid tumors. Thus, engineering tumor-reactive T cells resistant to immunosuppressive factors in the TME offers great potential. Here, we provide multiple lines of evidence—from gene expression to protein oxidation levels—that co-expression of the antioxidant TRX1 improves CAR T cell functions under pro-oxidative conditions. Collectively, our results indicate that TRX1 co-expression in CAR T cells can increase their resistance against the pro-oxidative TME and present a promising approach for the empowerment of CAR T cell therapies against solid tumors.

## Data availability statement

The MS-datasets presented in this study can be found in online repositories. The names of the repository/repositories and accession number(s) can be found below: http://proteomecentral.proteomexchange.org/, PXD031073.

## Ethics statement 

The isolation of peripheral blood mononuclear cells and their respective use for this study was approved by the Ethics Committee of Heidelberg University (S-089/2015).

## Author contributions

Conceptualization: EB and YS. Methodology: EB, HK, JL, CO, HA, TR, BN and KM-D. Investigation: EB, HK, NJ, DL, and VT. Resources: YS. Drafting of the article: EB and YS. Critical revision of the article: EB, CO, NJ, DL, JL, and YS. Supervision: YS. Funding acquisition: YS. All authors have approved the final version of the manuscript.
