# Supplementary material for: Expression of TRX1 optimizes the antitumor functions of human CAR T cells and confers resistance to a pro-oxidative tumor microenvironment
Source: Front Immunol. 2022 Dec 14;13:1063313. doi: 10.3389/fimmu.2022.1063313 (PMC9794734; doi:10.3389/fimmu.2022.1063313)
Supplement: Supplementary file 1 [file DataSheet_1.pdf]

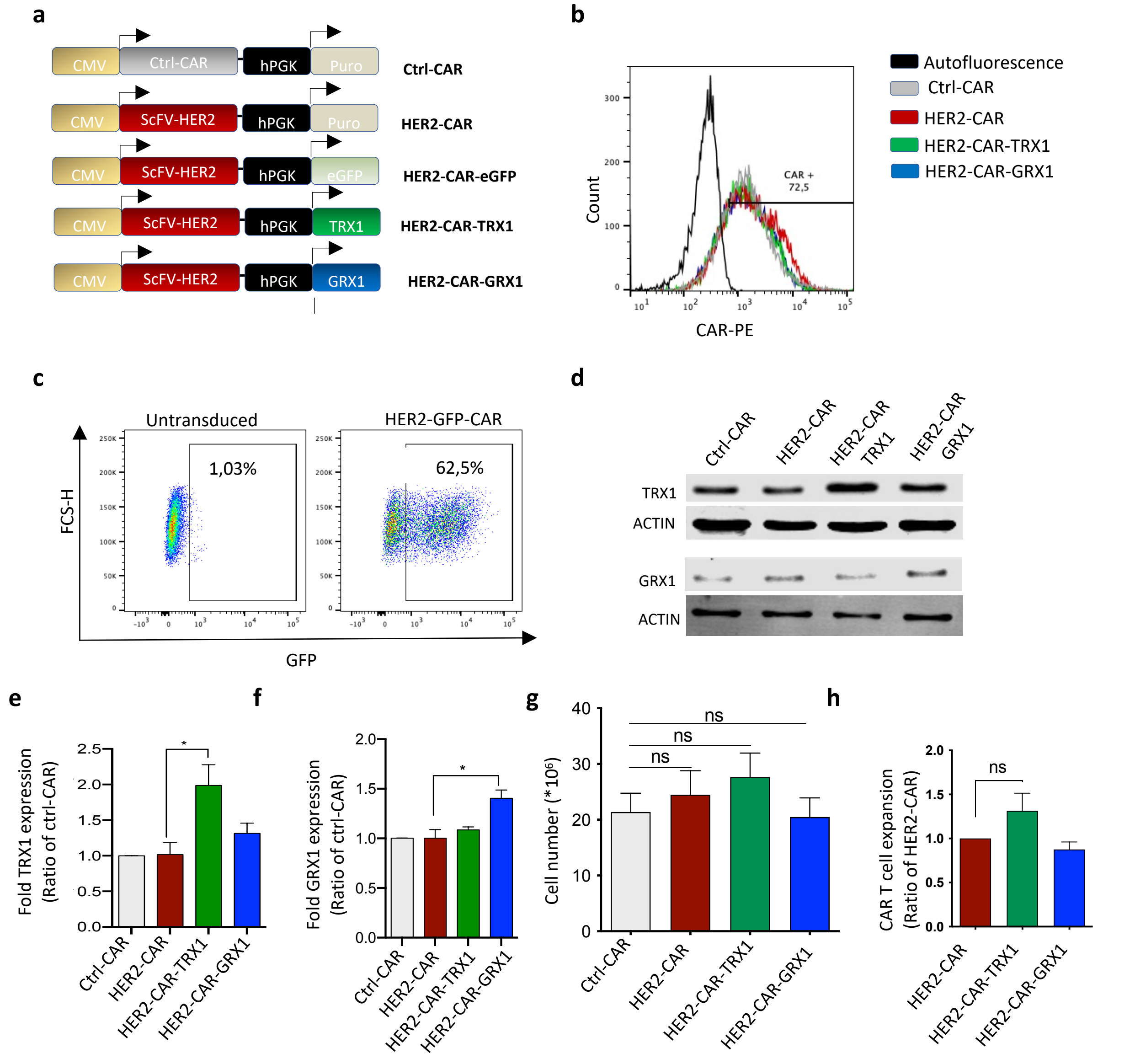

Supplementary Figure S1. Generation and characterization of CAR T cells with antioxidant empowerment. PBTs were activated with CD3/CD28 coated beads for one day. Then the cells were transduced with lentiviral particles carrying CAR constructs for three days. Thereafter, CAR T cells were cultured in culture medium and were characterized 8 days after activation. **(a)** Design of lentiviral vectors encoding for HER2-specific CAR construct and different antioxidant genes or eGFP. **(b)** Representative flow cytometry histogram showing expression of the CAR construct on activated T cells. **(c)** A representative flow cytometry pseudo-plots showing the expression of eGFP in activated T cells. **(d)** Representative immunoblots showing GRX1 and TRX1 expression in CAR T cells. Eight-day expanded CAR T cells were counted and equal amounts were lysed and loaded on SDS-polyacrylamide gels. Thereafter, samples were immunoblotted and stained for TRX1, GRX1, and ACTIN. (n=3). **(e, f)** Analysis of **(e)** TRX1- and **(f)** GRX1 expression in CAR T cells. The data are presented as the mean ± SEM (n ≥ 3; **p* < 0.05, ns = non-significant). P-values were calculated by t-test. **(g, h)** Expansion capacity of CAR T cells 8 days upon activation. **(g)** CAR T cell numbers 8 days after activation. **(h)** Ratio of HER2-CAR-TRX1 and HER2-CAR-GRX1 T cell numbers to HER2-CAR T cells 8 days after activation. The data are presented as the mean ± SEM (n = 8; **p* < 0.05, ns = non-significant).

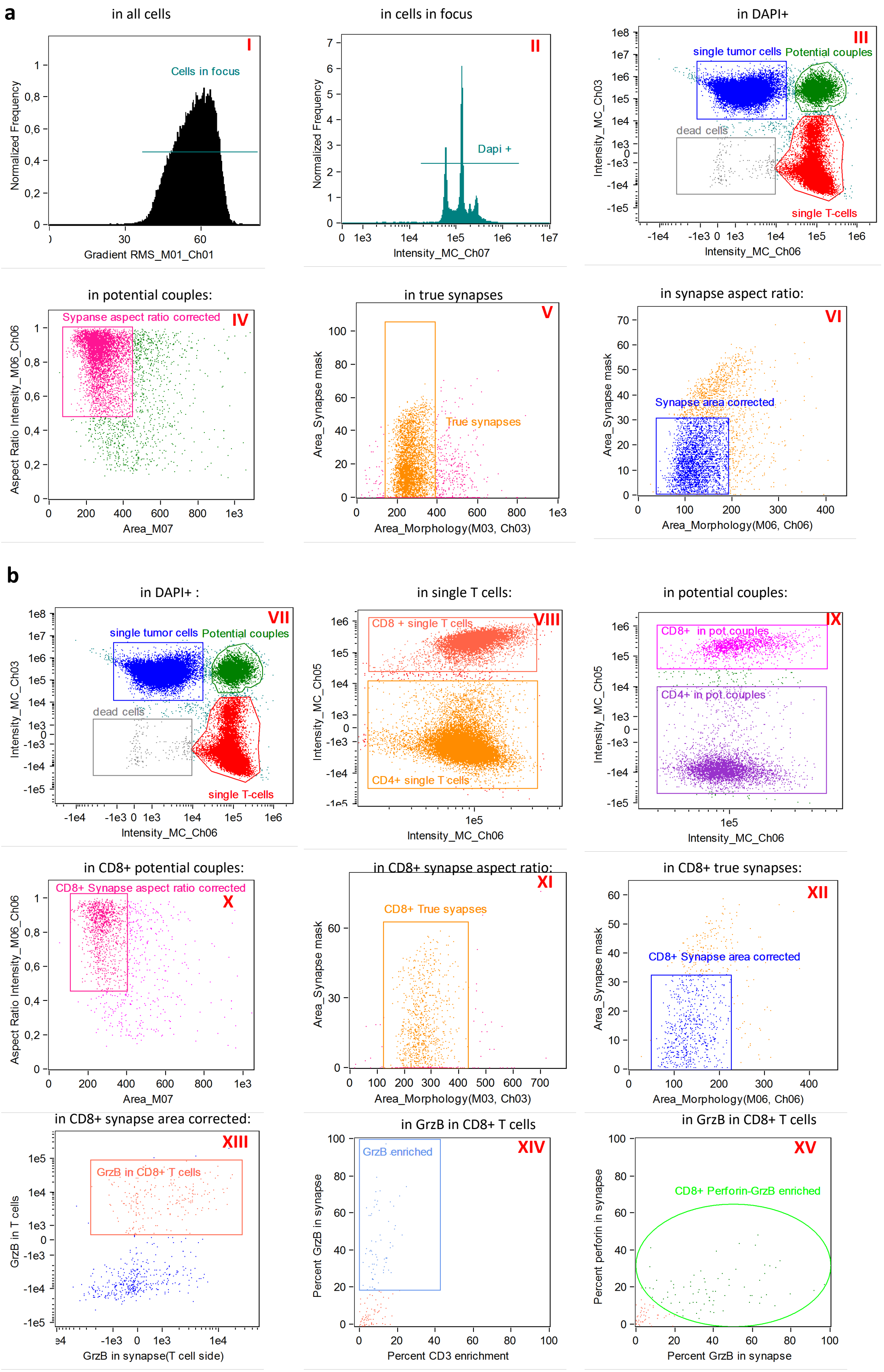
a
in all cells
I
Cells in focus
Normalized Frequency
Gradient RMS_M01_Ch01
in cells in focus
II
Dapi +
Normalized Frequency
Intensity_MC_Ch07
in DAPI+
III
single tumor cells
Potential couples
dead cells
single T-cells
Intensity_MC_Ch03
Intensity_MC_Ch06
in potential couples:
Sypanse aspect ratio corrected
IV
Aspect Ratio Intensity_M06_Ch06
Area_M07
in true synapses
V
True synapses
Area_Synapse mask
Area_Morphology(M03, Ch03)
in synapse aspect ratio:
VI
Synapse area corrected
Area_Synapse mask
Area_Morphology(M06, Ch06)
b
in DAPI+ :
VII
single tumor cells
Potential couples
dead cells
single T-cells
Intensity_MC_Ch03
Intensity_MC_Ch06
in single T cells:
VIII
CD8 + single T cells
CD4+ single T cells
Intensity_MC_Ch05
Intensity_MC_Ch06
in potential couples:
IX
CD8+ in pot.couples
CD4+ in pot.couples
Intensity_MC_Ch05
Intensity_MC_Ch06
in CD8+ potential couples:
CD8+ Synapse aspect ratio corrected
X
Aspect Ratio Intensity_M06_Ch06
Area_M07
in CD8+ synapse aspect ratio:
XI
CD8+ True syapses
Area_Synapse mask
Area_Morphology(M03, Ch03)
in CD8+ true synapses:
XII
CD8+ Synapse area corrected
Area_Synapse mask
Area_Morphology(M06, Ch06)
in CD8+ synapse area corrected:
XIII
GrzB in CD8+ T cells
GrzB in T cells
GrzB in synapse(T cell side)
in GrzB in CD8+ T cells
XIV
GrzB enriched
Percent GrzB in synapse
Percent CD3 enrichment
in GrzB in CD8+ T cells
XV
CD8+ Perforin-GrzB enriched
Percent perforin in synapse
Percent GrzB in synapse

Supplementary Figure S2 Gating strategy of cytolytic immune synapses between CAR T cells and SKBR3 cells. **(a)** Briefly, cells that were out of focus were gated out using Gradient RMS feature (region I). Next, cells that were stained for DAPI (Nuclei) were gated (region II). Following this, single T cells (CD3+ HER2-), single tumor cells (CD3- HER2+) and potential T cell and tumor cell couples (CD3+ HER2+) were discriminated based on CD3 and HER2 signals (region III). Next, using aspect ratio of CD3 signal intensity and the area of the nuclei, potential couples were further corrected (region IV). Cells which formed multiplets were gated out. Thereafter, a gate called „True synapses“ was generated based on the area of the synapse mask (Area_Synapse mask) and based on the area of tumor cells (Area_Morphology (M03, Ch03)) (region V). Next, „synapse area corrected“ gate was generated based on the area of the synapse mask (Area_Synapse mask) and the area of the T cell signal (Area_Morphology (M06, Ch06)) (region VI). These two consecutive gates allowed to define the tumor cell - T cell doublets more stringently by discriminating false positive interactions and multiplets.

(b) A similar gating strategy, as the strategy described above strategy was followed to discriminate CD4+ CAR T cells and CD8+ CAR T cells which were in contact with tumor cells. Shown is the gating strategy for CD8+ CAR T cells. Therefore, potential couples, single T cells, and single tumor cells were discriminated (region VII). Then, based on CD3 and CD8 staining, CD8+ CAR T cells and CD4+ CAR T cells were gated (region VIII- IX). CD3+ CD8- were considered as CD4+. Thereafter, CAR T cell and tumor cell couples were further corrected as described above (region X-XII). Next, granzyme B (XIII or granzyme A (not shown)) positive CD8+ or CD4+ T cells were gated. In this population, the translocation of granzyme B, CD3, and perforin into the interaction zone was calculated (XIV-XV).

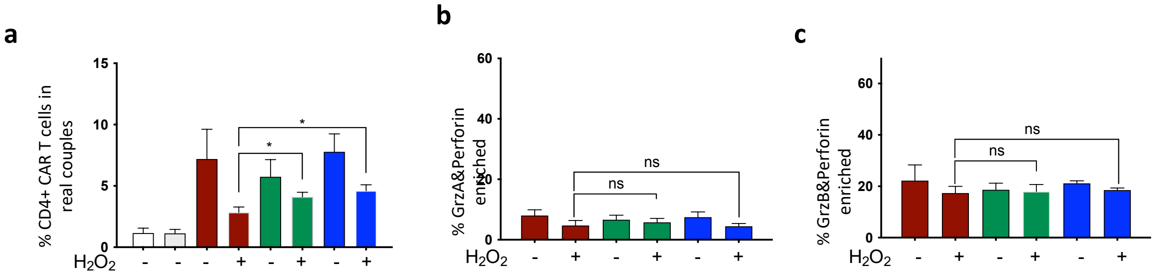

Supplementary Figure S3 Analysis of immune synapses between CD4+ CAR T cells and MDA-MB-453 cells. (a) % CD4+ CAR T cells in synapses with MDA-MB-453 tumor cells. **(b, c)** Quantification of enrichment of **(b)** Granzyme A and perforin, and **(c)** Granzyme B and perforin at the immune synapse. Data represent means ± SEM (n ≥ 3; *$p < 0.05$, ns = non-significant).

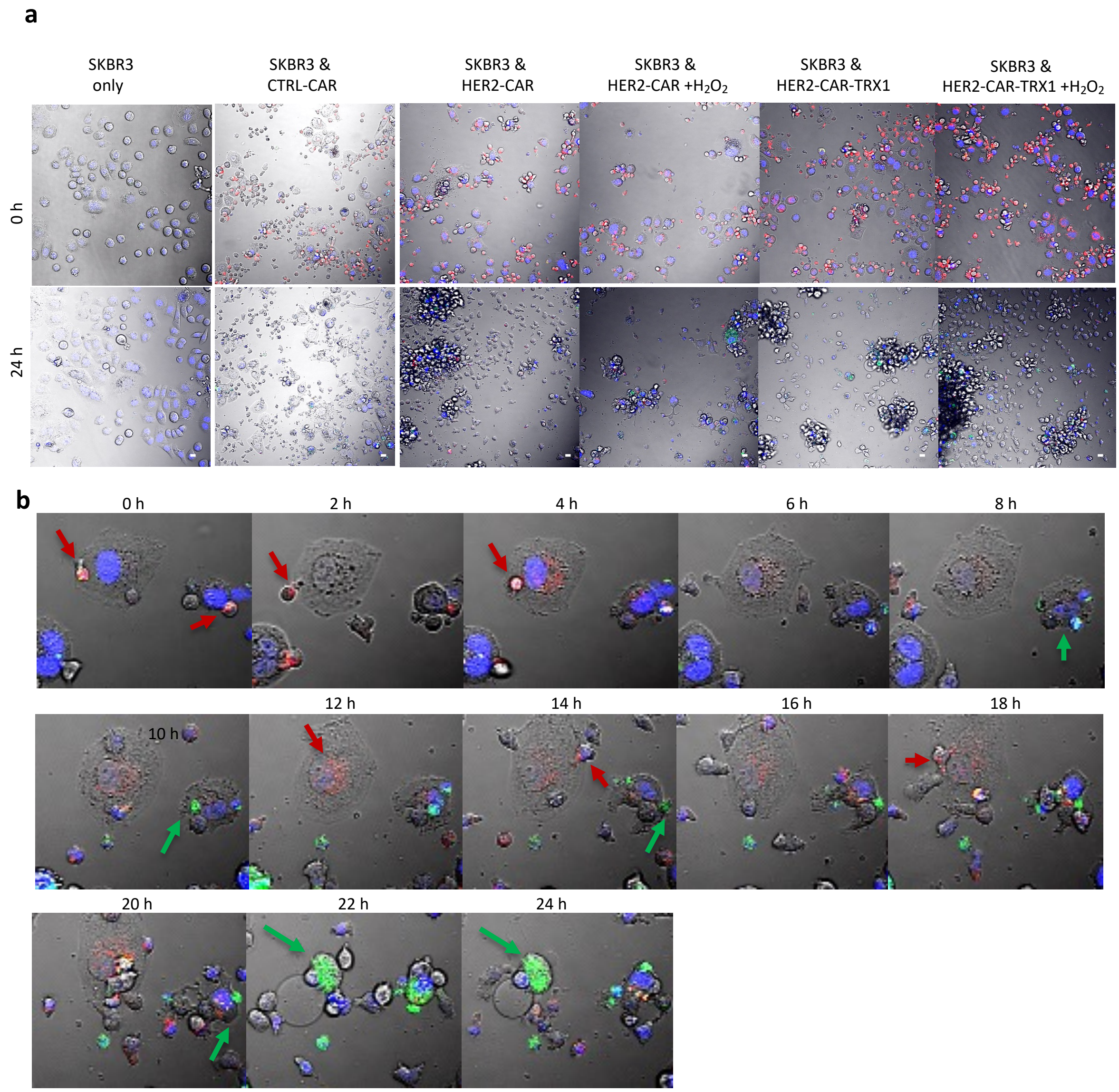

Supplementary Figure S4. CAR T cells efficiently kill target tumor cells over time. (a) Representative time-lapse images of CAR T and SKBR3 tumor cells at 0 and 24 h. Red (Lysotracker red), green (caspase 3/7 sensor), and blue (DAPI). SKBR3 cells were seeded on 8-well chambers and pre-stained with caspase 3/7 sensor and DAPI. CAR T cells were pre-stained with Lysotracker red. Then, CAR T cells were added onto SKBR3 tumor cells, and time-lapse imaging was performed for 26 h. Transmission light, DAPI (nuclei, blue), Lysotracker red (cytolytic granules, red), and caspase3/7 sensor (dying cells, green) are shown. Scale bar, 20 μm,(n = 3). **(b)** Time-lapse imaging of CAR T cell-mediated killing of SKBR3 cells performed using a Nikon laser scanning confocal microscope (NA 1.49). Red arrows, release of cytolytic granules from CAR T cells to tumor cells. Green arrows, dying cells (caspase 3/7 sensor); signal appears at 6 h. Tumor cell death was visible (cell shrinkage, appearance of caspase3/7 signal, and cell dwelling) from 16 h (n = 3).

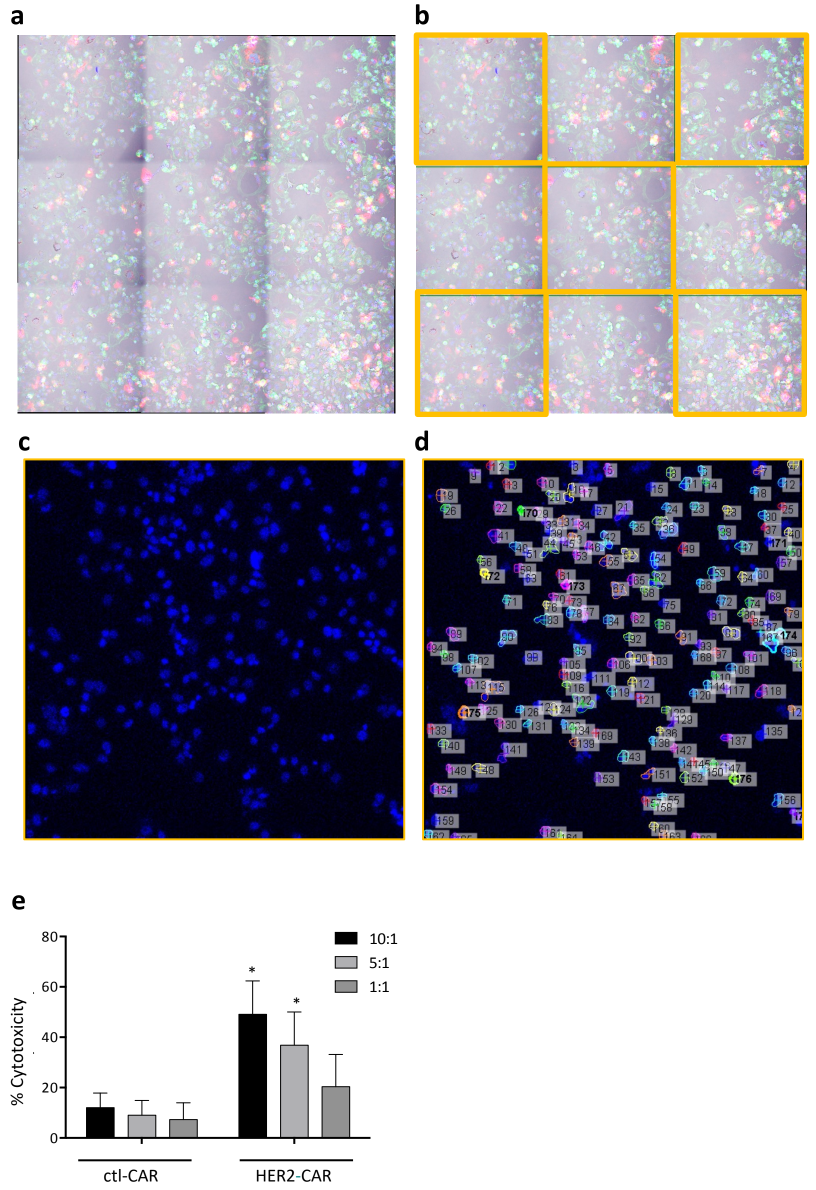

Supplementary Figure S5 Workflow of microscopy-based cytotoxicity assay. After coculture of eF607 prestained SKBR3 cells and CAR T cells, samples were fixed and stained for Nuclei (dapi, blue) and F-actin (phalloidin, green). **(a)** Then confocal images were acquired as 3xy large scans using the 10 x objective. **(b)** Next, image splitting was performed using NIS elements, resulting in nine single xy scans. Five of nine splitted image areas are shown with an orange square. **(c)** Shown is the 405 channel (DAPI) of one xy focal plane. **(d)** Using the automated region of interest (ROI) feature, SKBR3 tumor cells in each focal plane were counted based on intensity and area features of DAPI, and eF607 signals. Each number represents automatically counted SKBR3 tumor cells in the respective focal plane. (**e**) LDH release assay showing percentage of cytotoxicity on tumor cells in the presence of indicated CAR T cell-tumor cell ratios. Data represent means ± SEM (n ≥ 3; *$p < 0.01$).

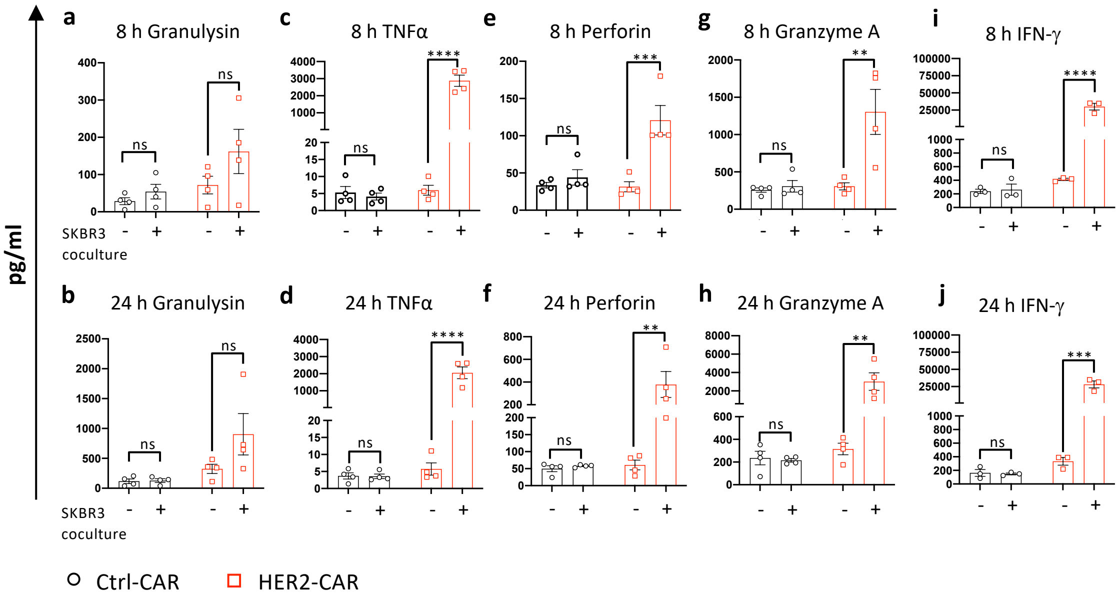

Supplementary Figure S6 Coculture with SKBR3 tumor cells leads to increase of cytokine production in HER2-CAR T cells. Ctrl-CAR and HER2-CAR T cells were cultured either alone (-) or in coculture with SKBR3 cells (+) (5:1 T cell/ tumor cell ratio). After 8 h and 24 h supernatants were collected and the release of cytokines was analysed using CD8/NK panel 13-plex. Shown are concentrations in pg/ml of Granulysin **(a)** 8 h and **(b)** 24 h, TNFα **(c)** 8 h and **(d)** 24 h, Perforin **(e)** 8 h and **(f)** 24 h, Granzyme A **(g)** 8 h and **(h)** 24 h, as well as IFN-γ **(i)** 8 h and **(j)** 24 h (n ≧ 3). Data represent means ± SEM. Statistical analysis was performed with two-way ANOVA (**p* < 0.05, ***p* < 0.01, ****p* < 0.001, *****p* < 0.0001, ns = non-significant).

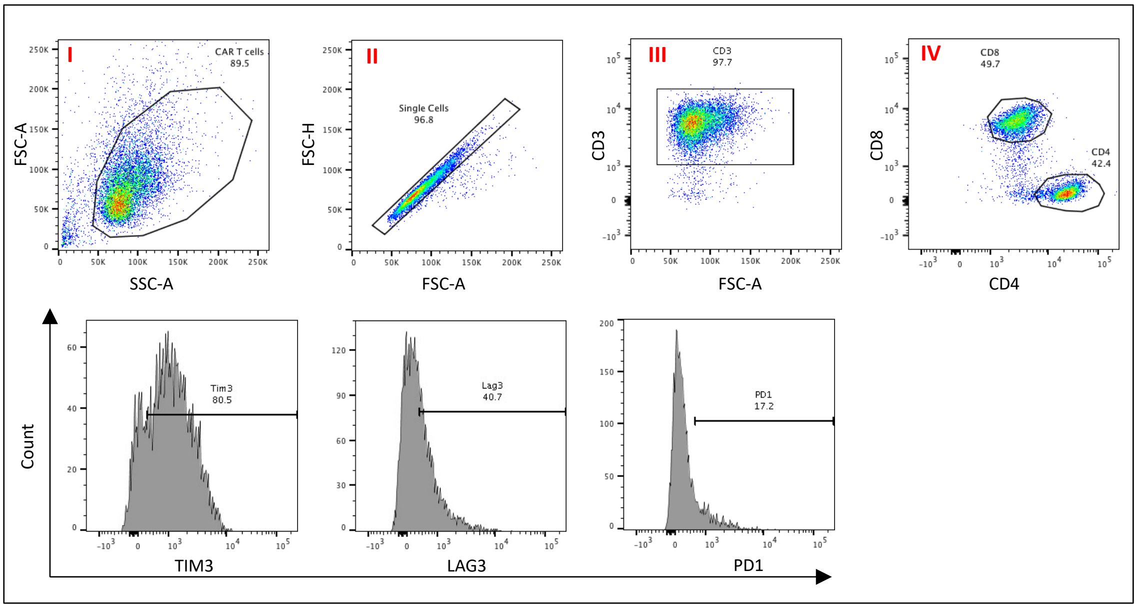

Supplementary Figure S7. Gating strategy to assess exhaustion state on CAR T cells. CAR T cells were generated and expanded for 8 days as described. Thereafter, the cells were fixed and stained for CD4, CD8, TIM3, LAG3 and PD1 markers to assess the exhaustion state of CD4+ and CD8+ CAR T cells. Cells were separated from debris based on SSC-A and FSC-A features (Region I). Next, single cells were gated using FSC-A and FSC-H features (Region II). CD3+ cells were gated (Region III). Thereafter, CD4+ and CD8+ CAR T cells were discriminated based on CD4 and CD8 staining (Region IV). In CD4+ and CD8+ CAR T cells, TIM3- (lower left histogram), LAG3- (lower middle histogram) and PD1-expression (lower right histogram) was assessed in CD4 and CD8+ CAR T cells.

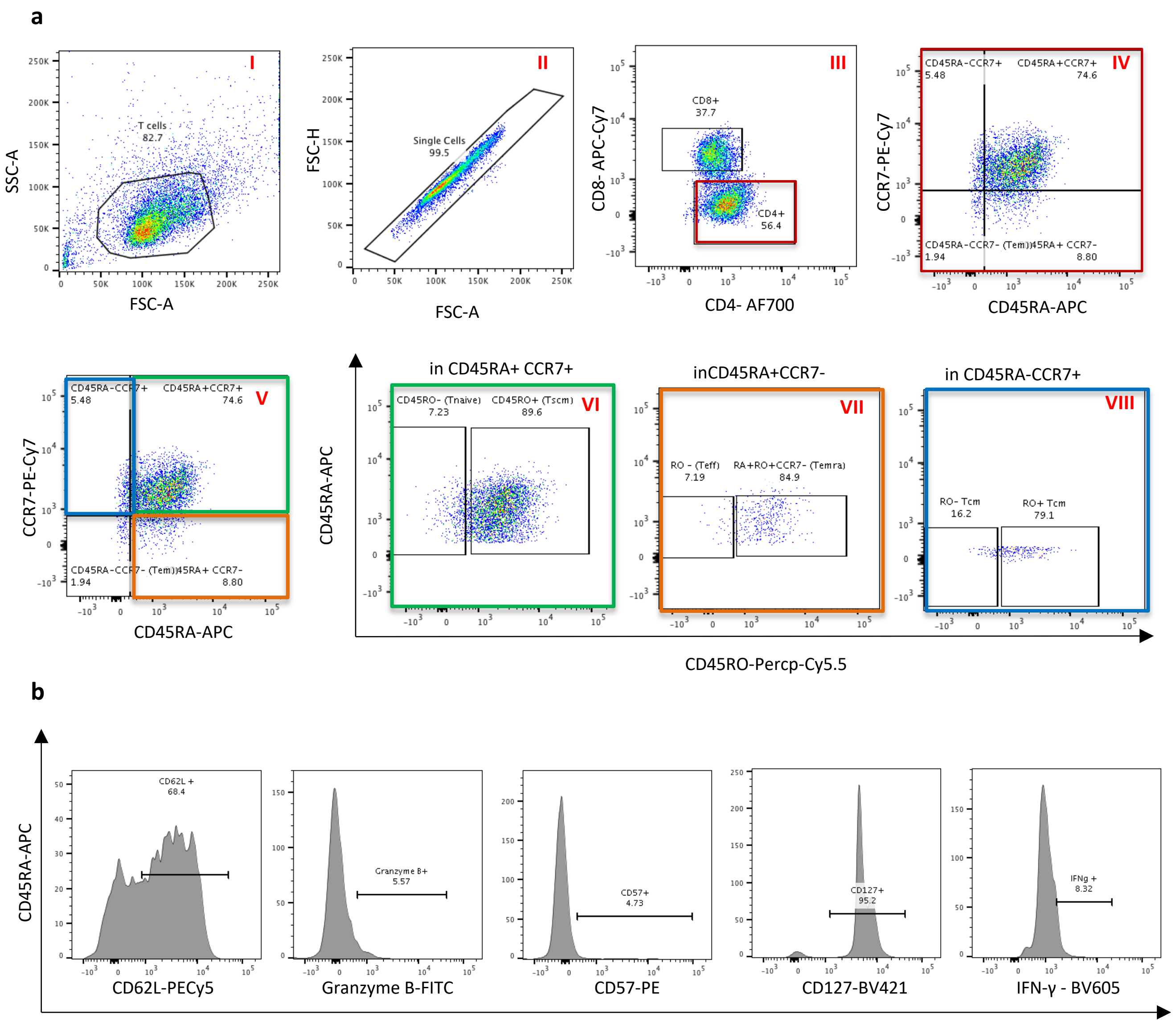

Supplementary Figure S8 (a) Gating strategy to assess memory state of HER2-CAR T cells. Cells were separated from debris based on SSC-A and FSC-A features (Region I). Next, single cells were gated using FSC-A and FSC-H features (Region II). Thereafter, CD4+ and CD8+ CAR T cells were discriminated based on CD4 and CD8 staining (Region III). Following that, CD45RA and CCR7 signals were used to discriminate memory phenotypes (Region IV and Region V). In CD45RA+ CCR7+ population, the CD45RO expression was assesed to discriminate Tscm (CD45RA+CCR7+CD45RO+) and Naive T cells (CD45RA+CCR7+CD45RO-). In CD45A+CCR7- negative population (Region VI), based on CD45RO expression, effector T cells (Teff) and RA+ effector memory T cells were discriminated (Temra) (Region VII). Next, in CD45RA-CCR7+ population, based on CD45RO expression, CD45RO+ Tcm and CD45RO-Tcm populations were discriminated. CD45RA-CCR7- population was considered as effector memory T cells. **(b)** Representative histogram graphs showing CD62L, Granzyme B, CD57, CD127 ad IFN-γ expression in CAR T cells.

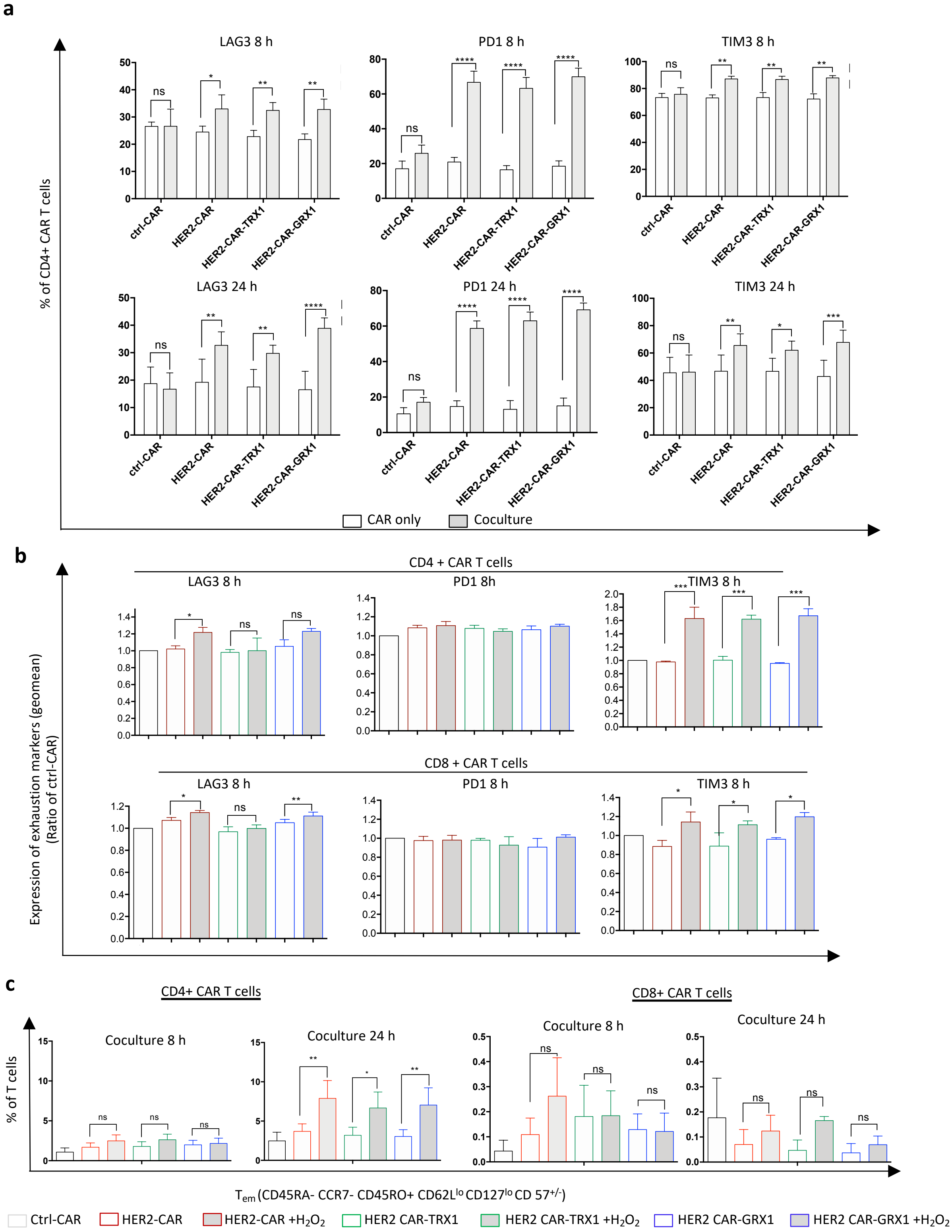
a
% of CD4+ CAR T cells
LAG3 8 h
PD1 8 h
TIM3 8 h
LAG3 24 h
PD1 24 h
TIM3 24 h
ctrl-CAR
HER2-CAR
HER2-CAR-TRX1
HER2-CAR-GRX1
CAR only
Coculture
b
Expression of exhaustion markers (geomean)
(Ratio of ctrl-CAR)
CD4 + CAR T cells
LAG3 8 h
PD1 8h
TIM3 8 h
CD8 + CAR T cells
LAG3 8 h
PD1 8 h
TIM3 8 h
c
CD4+ CAR T cells
CD8+ CAR T cells
% of T cells
Coculture 8 h
Coculture 24 h
Coculture 8 h
Coculture 24 h
T_{em} (CD45RA- CCR7- CD45RO+ CD62L^{lo} CD127^{lo} CD 57$^{+/-}$)
Ctrl-CAR
HER2-CAR
HER2-CAR +H_2O_2
HER2 CAR-TRX1
HER2 CAR-TRX1 +H_2O_2
HER2 CAR-GRX1
HER2 CAR-GRX1 +H_2O_2

Supplementary Figure S9 Analysis of the influence of antioxidant empowerment on the exhaustion and memory states of CAR T cells under control and pro-oxidative conditions. Ctrl-CAR T cells, HER2-CAR T cells, or antioxidant-empowered HER2-CAR T cells were kept untreated or treated with H_2O_2 and cocultured with SKBR3 cells for 8 h and 24 h. Thereafter, CAR T cells were collected and immunostained for exhaustion markers or markers discriminating memory phenotypes. **(a)** Percent expression of LAG3 (left), PD1 (middle) and TIM3 (right) on CD4+ CAR T cells after 8 h (upper graphs) and 24 h (lower graphs) of CAR only cultures (white bars) and CAR T cell - SKBR3 cocultures (grey bars). Data represent means ± SEM (n ≥ 3; * = p < 0.05, ** = p < 0.01, *** = p < 0.001, **** = p < 0.0001, ns = non-significant). **(b)** Expression of exhaustion markers (ratio of expression in ctrl-CAR T cells) in CD4+ and CD8+ HER2-CAR T cells that were cultured alone for 8 h**.** **(c)** Percent of CD4+ CAR T cells (left graphs) or CD8+ CAR T cells (right graphs) with T_{em} phenotypes in 8 h and 24 h CAR T cell-SKBR3 cocultures under control and pro-oxidative conditions. Data represent means ± SEM (n ≥ 3; * = p < 0.05, ** = p < 0.01, *** = p < 0.001, ns = non-significant).

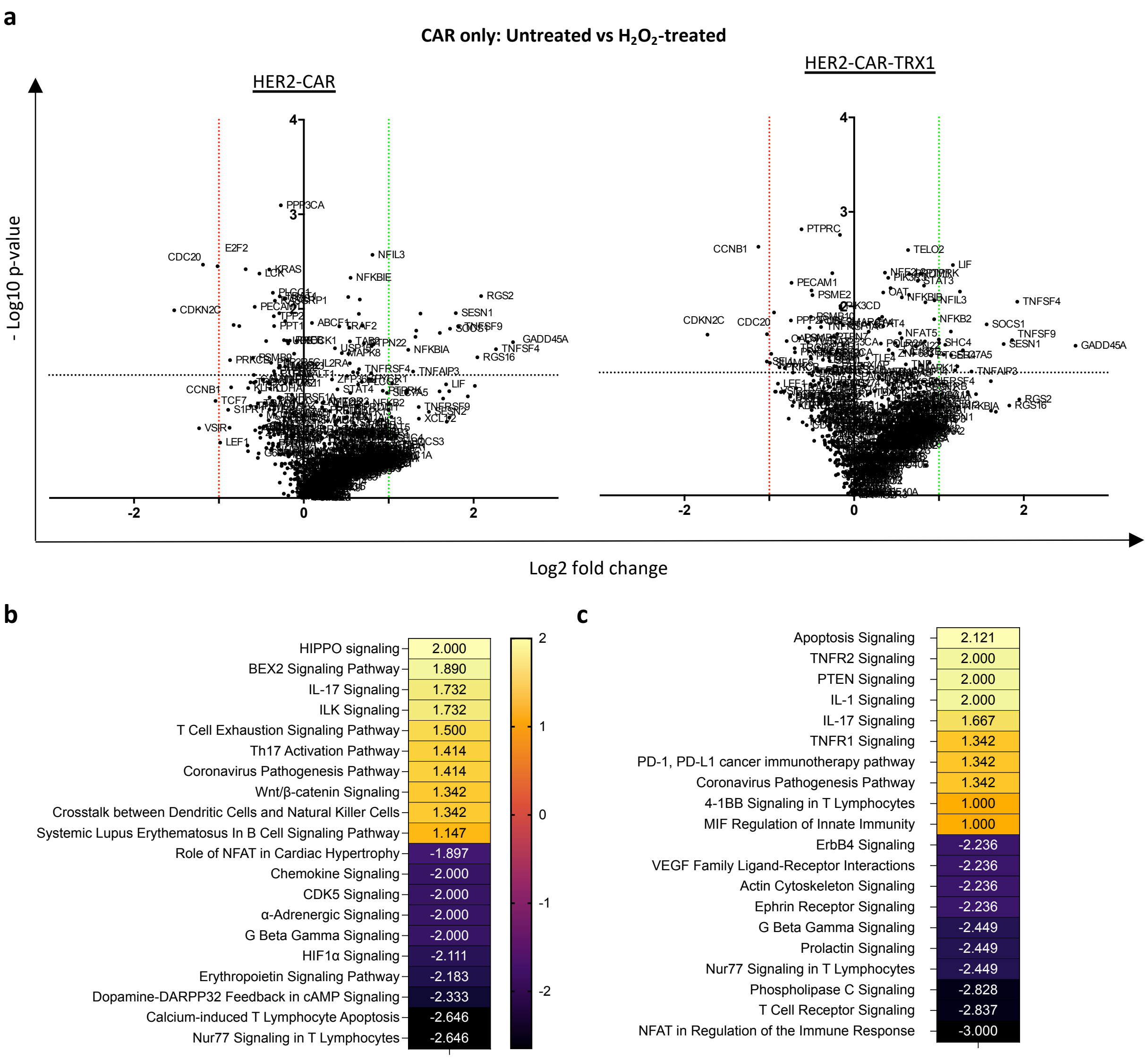

Supplementary Figure S10. **A pro-oxidative micromilieu alters the gene expression pattern of mRNAs in CAR T cells.** Ctrl-CAR T cells, HER2-CAR T cells and TRX1-empowered HER2-CAR T cells were kept untreated or treated with H_2O_2 for 1 h. The cells were then cultured alone. 5 h later, cells were harvested, total RNA was isolated and processed by nCounter mRNA analysis. **(a)** Volcano plot showing Log2 transformed mRNA expression under control vs pro-oxidative conditions in HER2 CAR T cells (left) and HER2-CAR-TRX1 T cells (right). **(b-c)** Ingenuity pathway (IPA) analysis of differentially regulated genes in Untreated vs H_2O_2-treated **(b)** HER2-CAR T cells and **(c)** HER2-CAR-TRX1 T cells underCAR only culture conditions.
